# Network-assisted genetic dissection of pathogenicity and drug resistance in the opportunistic human pathogenic fungus *Cryptococcus neoformans*

**DOI:** 10.1038/srep08767

**Published:** 2015-03-05

**Authors:** Hanhae Kim, Kwang-Woo Jung, Shinae Maeng, Ying-Lien Chen, Junha Shin, Jung Eun Shim, Sohyun Hwang, Guilhem Janbon, Taeyup Kim, Joseph Heitman, Yong-Sun Bahn, Insuk Lee

**Affiliations:** 1 Department of Biotechnology, College of Life Science and Biotechnology, Yonsei University, Seoul. 120-749, Korea; 2 Department of Biotechnology, Center for Fungal Pathogenesis, College of Life Science and Biotechnology, Yonsei University, Seoul. 120-749, Korea; 3 Department of Molecular Genetics and Microbiology, Medicine, and Pharmacology and Cancer Biology, Duke University Medical Center, Durham, North Carolina, USA; 4 Department of Plant Pathology and Microbiology, National Taiwan University, Taipei, Taiwan; 5 Institut Pasteur, Unité Biologie et Pathogénicité Fongiques, Département de Mycologie, F-75015, Paris, France

## Abstract

*Cryptococcus neoformans* is an opportunistic human pathogenic fungus that causes meningoencephalitis. Due to the increasing global risk of cryptococcosis and the emergence of drug-resistant strains, the development of predictive genetics platforms for the rapid identification of novel genes governing pathogenicity and drug resistance of *C. neoformans* is imperative. The analysis of functional genomics data and genome-scale mutant libraries may facilitate the genetic dissection of such complex phenotypes but with limited efficiency. Here, we present a genome-scale co-functional network for *C. neoformans*, CryptoNet, which covers ~81% of the coding genome and provides an efficient intermediary between functional genomics data and reverse-genetics resources for the genetic dissection of *C. neoformans* phenotypes. CryptoNet is the first genome-scale co-functional network for any fungal pathogen. CryptoNet effectively identified novel genes for pathogenicity and drug resistance using *guilt-by-association* and *context-associated hub* algorithms. CryptoNet is also the first genome-scale co-functional network for fungi in the basidiomycota phylum, as *Saccharomyces cerevisiae* belongs to the ascomycota phylum. CryptoNet may therefore provide insights into pathway evolution between two distinct phyla of the fungal kingdom. The CryptoNet web server (www.inetbio.org/cryptonet) is a public resource that provides an interactive environment of network-assisted predictive genetics for *C. neoformans*.

C*ryptococcus neoformans* is an opportunistic human pathogenic fungus. *C. neoformans* var. *grubii* (serotype A) and *C. neoformans* var. *neoformans* (serotype D) generally cause fatal meningoencephalitis in immunocompromised patients such as HIV/AIDS patients. In contrast, *Cryptococcus gattii* (formally known as *C. neoformans* var. *gattii* serotypes B and C) affects immunocompetent individuals^1^. Systemic cryptococcosis causes severe global mortality, with approximately 600,000 deaths per year^2^. Classical approaches have revealed two major virulence factors, a polysaccharide capsule^3^ and melanin^3^,^4^, which are distinguishable from most fungal pathogens. Although effective antifungal drugs are available, treatments of cryptococcosis often fail for several reasons, including antifungal drug resistance^5^. Novel therapeutics for the treatment of cryptococcosis are currently in high demand. Like other pathogenic fungi, the pathways for pathogenicity and antifungal drug resistance in *C. neoformans* remain elusive. *C. neoformans* requires a high level of integrity of its complex pathways to successfully infect the cells of a human host. A reconstruction of the pathways of pathogenicity and drug resistance in *C. neoformans* may provide new insights into antifungal treatments.

Systematic tools that accelerate the discovery of new genes for pathogenicity and drug resistance are needed to meet the urgent demand for new anticryptococcal treatments. Gene expression signatures from microarray or RNA-seq experiments have proven useful to investigate pathways that modulate pathogenicity and drug susceptibility^6^,^7^,^8^. The majority of expression responses, however, originate from indirect effects triggered when primary genes change their activity, which hampers the identification of the genes directly associated with the target pathways. In addition, not all cellular processes are regulated by gene expression, such as those that are subject to post-transcriptional regulation. Evidence from mutant phenotypes is generally more reliable and intuitive for identifying novel genes for virulence or drug resistance. Recently, a systematic knockout library of 1,201 *C. neoformans* genes became available, and was used to identify novel genes relevant to virulence^9^. This mutant library, however, covers only 20% of the *C. neoformans* genome. The construction of mutant strains for the remaining genes and the testing for each virulence-related phenotype would be prohibitively expensive and time-consuming.

Neither functional genomics data nor reverse genetics resources alone, therefore, can meet the current demand for efficient genetic dissection. Recently, several studies have suggested the use of gene networks as bridges between these two research resources. Co-functional gene networks have been shown to be effective in gene-to-phenotype mapping^10^,^11^,^12^. Genes that lie closer to one another in the network are highly likely to be involved in the same function or phenotype. This principle of guilt-by-association recently has grown in popularity for the identification of novel genes for a cellular function or phenotype. Previously, the network-assisted genetic dissection of complex phenotypes has proven effective in a model fungus, *Saccharomyces cerevisiae*, using a genome-scale co-functional gene network, YeastNet^13^,^14^. Because the genetic principles of complex phenotypes are similar across fungal species, the network-assisted approach may facilitate the effective identification of novel genes for virulence and adaptation to chemical stresses in pathogenic fungi, including *C. neoformans*, provided that a high quality co-functional gene network becomes available.

Although a few molecular networks for pathogenic fungi have been reported over the past several years, these networks were either small protein-protein interaction networks^15^,^16^,^17^ or transcriptional regulatory networks relevant to specific cellular conditions^18^,^19^. More recently, a genome-wide scale-free network of *Candida albicans* has been reported^20^, but its quality has been assessed by only a few network hub genes with no experimental validation. The network edge information and analysis tools for hypothesis generation are not available to the public for any of these networks, however, and therefore neither the reassessment nor the reuse of these networks is possible. The limited progress in the development of molecular networks for non-model pathogenic fungi are due in large part to the lack of experimental data. Nevertheless, this shortcoming may be partially overcome by the orthology-based transfer of gene networks from other species^21^,^22^. The transfer of potentially false links from other species can be minimized by the judicious weighting of links for pathogenic fungi.

In this work, we report a genome-scale co-functional network for *C. neoformans*, CryptoNet (www.inetbio.org/cryptonet), which was constructed by integrating 14 distinct types of large-scale data and covers ~81% of the coding genome. We find that CryptoNet is highly predictive for known *C. neoformans* virulence genes, and apply it to predict novel genes involved in virulence and drug response. Our results expand our view of the pathways relevant to fungal pathogenicity and drug resistance, which potentially may lead to the development of novel therapeutic targets.

## Results

### Construction of a genome-scale co-functional gene network for *C. neoformans*

**Figure 1:**
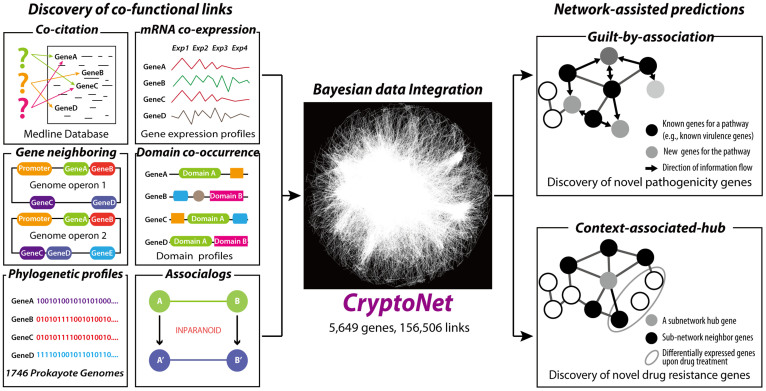
A summary of network-assisted genetics approaches to study *C. neoformans* pathogenicity and drug resistance. The co-functional links between *C. neoformans* genes derived from 14 diverse data sets, including co-citation, co-expression, domain co-occurrence, gene neighborhood, phylogenetic profiling, and associalogs from *S. cerevisiae* and *H. sapiens*, were integrated by Bayesian statistics into a single network, CryptoNet. Two network approaches, guilt-by-association and context-associated hub, were used to predict novel genes involved in pathogenicity and drug resistance in *C. neoformans*.

The network construction and network-assisted predictions for the *C. neoformans* gene network are summarized in Figure 1. To benchmark inferred co-functional links from large-scale data, we used gold standard gene pairs derived from the Kyoto Encyclopedia of Genes and Genomes (KEGG) pathway annotations^23^. These known links for the same metabolic pathways cover only 1,414 *C. neoformans* genes (i.e., only ~20% of all 6,975 coding genes). Log likelihood scores (LLS) based on a Bayesian statistical framework and weighted sum (WS) methods were used to integrate diverse types of data derived from three different species (*C. neoformans, S. cerevisiae*, and *H. sapiens*); these methods were used previously in the construction of the co-functional network for *S. cerevisiae*^13^.

**Figure 2:**
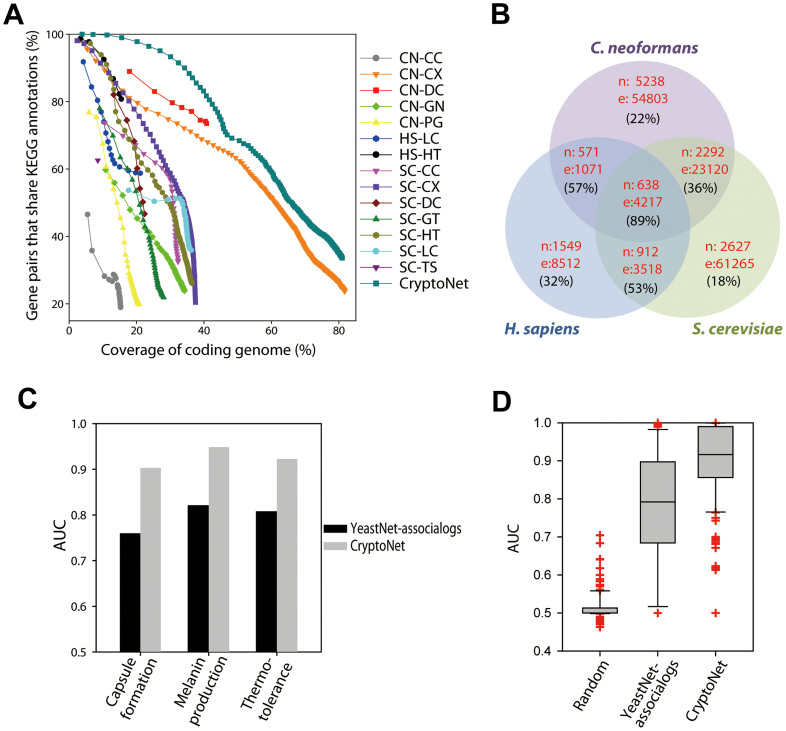
Assessment of CryptoNet. (A) A performance curve shows that CryptoNet outperforms all individual networks associated with each data type. The *x*-axis represents the percentage coverage of the *C. neoformans* coding genome and the *y*-axis represents the percentage of gene pairs that share KEGG pathway annotations. Each data point represents a bin of 1,000 co-functional links ordered by the log likelihood score (LLS). Data sets are named as XX-YY, where XX represents the origin species of data (CN, *C. neoformans*; HS, *H. sapiens*; SC, *S. cerevisiae*) and YY represents the data type (CC, co-citation; CX, co-expression; DC, domain co-occurrence; GN, gene neighborhood; GT, genetic interaction; HT, high throughput protein-protein interactions; LC, literature-curated protein-protein interactions; PG, phylogenetic profile similarity; TS, protein-protein interactions inferred from the tertiary structure). (B) The Venn diagram illustrates the overlap among three species-associated co-functional links in CryptoNet. The number of genes and links of the networks for each compartment of the diagram are also marked as ‘n:' followed by the node (i.e., gene) count and ‘e:' followed by the edge (i.e., link) count. The accuracy of each network, which is the percentage of correctly retrieved gene pairs that share KEGG pathway annotations, is also indicated in parentheses. (C) In a comparison of the AUC scores (i.e., network prediction power) between CryptoNet and a *C. neoformans* gene network derived from YeastNet for three virulence phenotypes, CryptoNet exhibits substantially improved predictive powers for all three virulence phenotypes. (D) For 162 UniProtGOA biological process terms (only terms with more than five annotated genes were considered), CryptoNet shows significantly higher range of AUC scores than that of YeastNet-associalogs (*p*-value < 2.2 × 10^−16^, Wilcoxon signed rank test), suggesting that the higher prediction power of CryptoNet can be generalized to many biological processes.

Co-functional associations between two genes were inferred from five distinct types of *C. neoformans* data: the probability of co-citation in Medline articles (CC), co-expression patterns across microarray samples (CX), the co-occurrence of protein domains (DC), phylogenetic profile similarity (PG), and orthologous gene neighborhoods in bacterial genomes (GN). In addition, orthologous functional associations (associalogs)^21^ were transferred from previously constructed co-functional networks for *S. cerevisiae*^13^ and *H. sapiens*^24^. More than half of the *C. neoformans* genes have orthologs in either *S. cerevisiae* or *H. sapiens*. A total of 14 networks derived from *C. neoformans* specific data and orthology-based transfer data were integrated into a single network of *C. neoformans* genes, CryptoNet, which maps 156,506 co-functional links among 5,649 genes (i.e., ~81% of coding genome). More details about the network construction are described in the Supplementary Methods. Information about the data sources and inference methods used for the co-functional links in CryptoNet are summarized in Supplementary Table S1. A benchmark of the networks using the percentage of gene pairs that share KEGG annotations with bootstrapping shows that the network developed through data integration improved the network quality both with respect to the genome coverage and the accuracy compared with those networks developed from individual data types (Figure 2A). The edge information for the integrated CryptoNet as well as all individual networks for the 14 distinct data types is available from the CryptoNet web server (www.inetbio.org/cryptonet/). The CryptoNet web server also provides three network search options for hypothesis generation (Figure S1): i) ‘*Find new members of the pathway*'; ii) ‘*Infer functions from network neighbors*'; and iii) ‘*Find context-associated hub genes*'. All predictions made in this study were generated by the network search tools from the CryptoNet web server.

### *C. neoformans* specific-data are critical for the prediction of fungal pathogenicity

Many co-functional links in CryptoNet were derived from *S. cerevisiae* and *H. sapiens* data. To examine the extent to which genomic information derived from *C. neoformans* contributes to the quality of the integrated network, we divided the CryptoNet links into three species-associated networks, and then measured the accuracy of each network and their intersections using the percentage of gene pairs that share KEGG annotations (Figure 2B). The most accurate network was the one conserved among all three species (89%), followed by those conserved between two species (57% for the network conserved between *C. neoformans* and *H. sapiens*, 53% for the network conserved between *S. cerevisiae* and *H. sapiens*, and 36% for the network conserved between *C. neoformans* and *S. cerevisiae*) and species-specific links (32% for *H. sapiens*-specific links, 22% for *C. neoformans*-specific links, and 18% for *S. cerevisiae*-specific links). The observed hierarchy of network accuracy indicates that the integration of co-functional links derived from multiple species generally improves the quality of the *C. neoformans* gene network.

Next, we examined the predictive power of CryptoNet for *C. neoformans* pathogenicity. The assessment of predictive power requires known pathogenicity genes. We collected 73 virulence genes for three different virulence phenotypes from the literature: 28, 23, and 22 genes for capsule formation, melanin production, and thermotolerance, respectively (Supplementary Table S2). A popular method of gene prioritization is the guilt-by-association principle (see Figure 1). We used a sophisticated version of guilt-by-association that takes into account the weights on the network edges. The ranks of the virulence genes were assigned by the sum of the edge scores (LLS) to all virulence genes. We then assessed the predictive power of CryptoNet for each of the three virulence phenotypes using a receiver operating characteristic (ROC) analysis, the results of which were summarized by the area under the ROC curve (AUC) (see Supplementary Methods). If the known virulence genes are highly interconnected within CryptoNet, then these genes become highly ranked using this network-assisted prioritizing method. Using the ‘*Find new members of the pathway*' search option from the CryptoNet web server, we found that 73 query genes for each of the three virulence phenotypes were highly interconnected, which resulted in high AUC scores: 0.9031, 0.9483, and 0.9226 for capsule formation, melanin production, and thermotolerance, respectively (Figure 2C). These results suggest that CryptoNet is highly predictive for *C. neoformans* pathogenicity.

*S. cerevisiae* has been used as a reference species for fungal pathogens, including *C. neoformans*. A co-functional network of *S. cerevisiae*, YeastNet v3^13^, was transferred into CryptoNet. YeastNet v3 comprised ~59% (92,120 of 156,506) of the CryptoNet links. To determine the extent to which the non-yeast-derived links contribute to the observed predictability for pathogenicity, we compared the predictability for the three *C. neoformans* virulence phenotypes between a YeastNet-derived network (i.e., YeastNet-associalogs) and CryptoNet. We found significantly reduced AUC scores for the YeastNet-derived network: 0.7598, 0.8201, and 0.8068 for capsule formation, melanin production, and thermotolerance, respectively (Figure 2C). This result indicates that genomics data from *C. neoformans* and *H. sapiens* contribute substantial information to *C. neoformans* pathogenicity. By performing assessments of predictability for 162 UniProtGOA terms^25^, we also found that the superior prediction power of CryptoNet to the YeastNet-derived network is not limited to the three virulence phenotypes, but can be generalized to many biological processes (Figure 2D). Notably, the network information of 2,536 CryptoNet genes (~47% of the coding genes) was derived from *C. neoformans*-specific data only, which suggests that *C. neoformans*-specific data contributes significantly to the high genome coverage of CryptoNet. Importantly, *C. neoformans* belongs to the basidiomycota phylum. In contrast, many other popular laboratory fungi, including *S. cerevisiae*, belong to the ascomycota phylum. *S. cerevisiae* data are not expected, therefore, to be sufficient for the study of *C. neoformans*, which belongs to a distant phylogeny group. To the best of our knowledge, CryptoNet is the first genome-scale gene network for a fungal species in the basidiomycota phylum. CryptoNet may therefore provide insights into pathway evolution between two distinct phyla of the fungal kingdom.

### CryptoNet identifies novel genes for *C. neoformans* pathogenicity

The effective retrieval of known virulence genes by CryptoNet suggests that other highly ranked genes are also likely involved in the virulence of *C. neoformans*. We therefore chose the top 100 candidate genes for each of the three virulence phenotypes, and performed assays of virulence factor production and thermotolerance with available deletion mutant strains from the Madhani collection^9^, which was obtained from the Fungal Genetics Stock Center (Supplementary Table S3).

**Figure 3:**
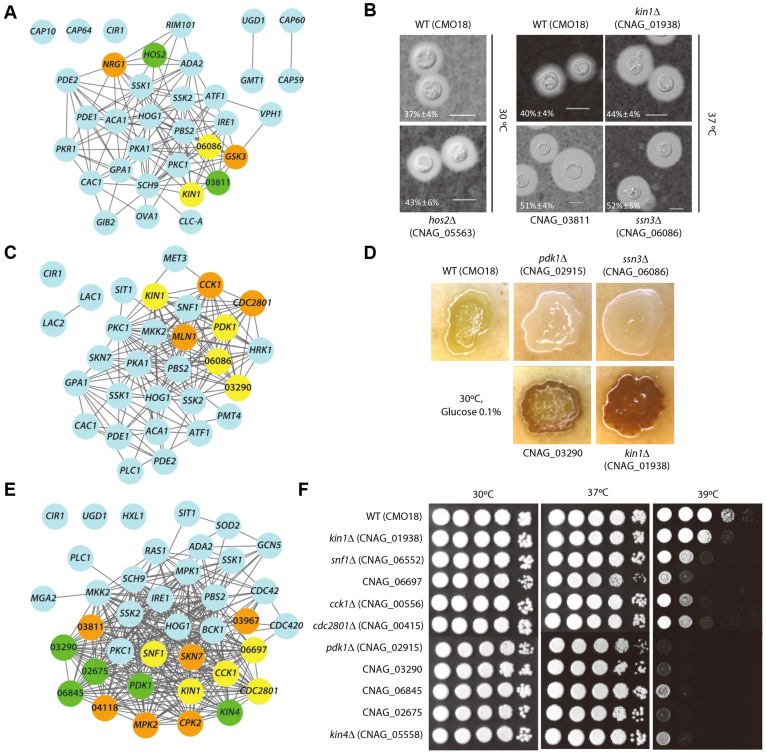
Network-assisted identification of novel genes for three virulence phenotypes. The cyan-colored nodes represent 73 genes known to be associated with the three virulence phenotypes. The yellow-, orange-, and green-colored nodes indicate virulence genes that were identified in our study, in Madhani's study^9^, and in both studies, respectively. (A) The 28 known genes and 6 novel genes associated with capsule formation. (B) The capsule formation assay results. The overnight cell culture (5 μl) was spotted onto the agar-based DME medium and further incubated for two days at either 30°C or 37°C. The capsules were visualized by India ink staining and photographed using an Olympus BX51 microscope equipped with a SPOT insight digital camera. Quantitative measurement of capsule size was determined by measuring the ratio of capsule size to the cell size. The scale bar represents 10 μm. Quantitative measurement of capsule size was determined by measuring the ratio of capsule size to the cell size (indicated as percentage). (C) The 23 known genes and seven novel genes associated with melanin production. (D) The cells were spotted onto Niger seed medium containing 0.1% glucose, incubated at 30°C, and photographed after 4 days. (E) The 22 known genes and 16 novel genes associated with thermotolerance. (F) Each strain that grew to the mid-logarithmic phase was 10-fold serially diluted and spotted on YPD medium, and further incubated at either 30°C, 37°C or 39°C for the thermotolerance assay.

Capsule production assays validated four of the 40 tested strains by either Madhani's study or our study (discovery rate = 10%, Figure 3A): CNAG_03811, CNAG_01938 (*KIN1*), CNAG_05563 (*HOS2*), and CNAG_06086 (*SSN3*, a yeast ortholog). Although all four of these mutant strains exhibited increased capsule production compared with the parental CMO18 strain in our study (Figure 3B), *ssn3*Δ and *kin1*Δ mutant strains were tested but reported not to enhance capsule production in Madhani's study^9^. This difference in results may be attributed to the different experimental conditions between the two studies. For example, we used a capsule-inducing medium (Dulbecco's modified Eagle solid medium) that was different from the one used for Madhani's study (10% Sabouraud medium buffered to pH 7.3 with 50 mM MOPS). The relative capsule diameter was determined by measuring the ratio of the capsule size to the cell size (Figure 3B). Two more genes, CNAG_05222 (*NRG1*) and CNAG_06730 (*GSK3*), were reported to regulate capsulate production in Madhani's study, but were not testable in our study due to a failure in strain recovery. Another independent study has reported that *NRG1* is involved in capsule formation^26^.

We next validated four of the 36 tested strains for melanin production by either study (discovery rate = 11.11%, Figure 3C). Four deletion strains for CNAG_02915 (*PDK1*), CNAG_03290, *SSN3*, and *KIN1* showed altered levels of melanin production compared with the CMO18 strain (Figure 3D). Notably, all of these strains were tested but reported not to affect melanin production in Madhani's study^9^. This difference also may result from different experimental conditions between the two studies. For example, we used a melanin-inducing medium (Niger seed medium) that was different from the one used for Madhani's study (L-DOPA medium). There were also three deletion strains that showed altered melanin production in Madhani's study but not in this study: CNAG_00415 (*CDC2801*), CNAG_00556 (*CCK1*), and CNAG_04837 (*MLN1*).

To validate the predictions for genes that contribute to thermotolerance, the growth of 37 candidate genes in the Madhani collection was monitored at 30°C, 37°C, or 39°C. Ten genes were shown to be involved in growth at high temperatures (37°C or 39°C, discovery rate = 27.03%, Figure 3E). Six additional strains were reported to contribute to thermotolerance in Madhani's study but were not tested in this study: CNAG_02531 (*CPK2*), CNAG_03409 (*SKN7*), CNAG_03967 (*CAP1*, a yeast ortholog), CNAG_04118 (*CTK1*), CNAG_04282 (*MPK2*), and CNAG_03811. Five of the 10 tested strains, CNAG_02675 (*HSL1*, a yeast ortholog), *PDK1*, CNAG_03290, CNAG_05558 (*KIN4*), and CNAG_06845 (*CDC15*, a yeast ortholog), were also reported in Madhani's study to show grow defects at 37°C^9^. The roles of CNAG_06552 (*SNF1*) and *CCK1* in thermotolerance have been reported in the serotype D JEC21 and the serotype A H99 strain backgrounds, respectively^27^,^28^,^29^. The current study identified three new genes involved in thermotolerance: *CDC2801*, CNAG_06697 (*MPS1*, a yeast ortholog), and *KIN1* (Figure 3F).

**Figure 4:**
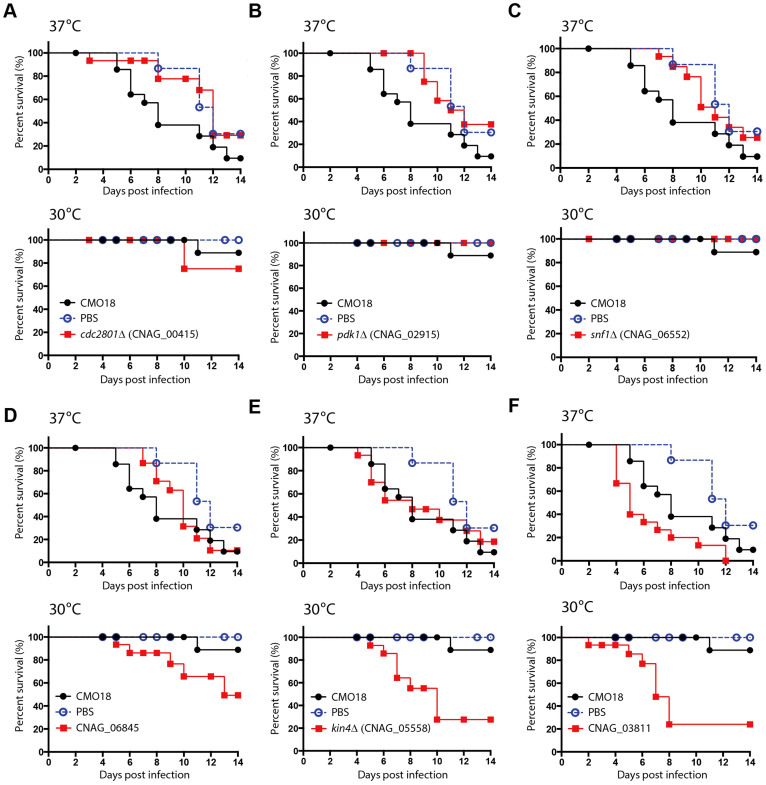
*In vivo* virulence assay of candidate genes using a wax moth model system. Each larva (15 per group) was infected with 800,000 *Cryptococcus* cells and incubated at either 30°C or 37°C. The percent survival (%) was monitored for two weeks post-infection. The results were as follows: *p* = 0.0337 for CMO18 vs. the *cdc2801*Δ (CNAG_00415) mutant at 37°C; *p* = 0.0064 for CMO18 vs. the *pdk1*Δ (CNAG_02915) mutant at 37°C; *p* = 0.0269 for CMO18 vs. the *snf1*Δ (CNAG_06552) mutant at 37°C; *p* = 0.0394 for CMO18 vs. CNAG_06845 at 30°C; *p* = 0.0021 for CMO18 vs. the *kin4*Δ (CNAG_05558) mutant at 30°C; *p* = 0.007 and *p* = 0.0155 for CMO18 vs. CNAG_03811 at 30°C or 37°C, respectively.

Based on the virulence factor phenotypes, we performed an additional *in vivo* virulence assay with 13 strains that showed an altered virulence factor formation using a wax moth model (*Galleria mellonela*) (Figure 4 and Supplementary Figure S2). Among these 13 strains, three deletion strains for *CDC2801*, *PDK1*, or *SNF1* exhibited attenuated virulence at 37°C (Figure 4A–C), which reflects the growth defects of these strains at higher temperatures (Figure 3F). These results agree with the previously reported signature tagged mutagenesis (STM)-based mouse data^9^. In contrast, two mutant strains (deletion of either *CDC15* or *KIN4*) exhibited increased virulence at 30°C but not at 37°C (Figure 4D and E). Notably, the deletion of CNAG_03811 was shown to enhance capsule production (Figure 3B) and induce hypervirulence in the insect infection model at both 30°C and 37°C (Figure 4F).

To further validate CryptoNet, we deleted three candidate genes, *KIN1*, CNAG_05499 (*SHO1*, a yeast ortholog) and CNAG_04678 (*YPK1*), that were predicted by CryptoNet to be involved in virulence factor formation, and tested their *in vitro* and *in vivo* phenotypes. First, we constructed two independent *kin1*Δ mutants in the serotype A H99S strain to confirm the altered virulence factor formations in the *kin1*Δ mutant on a CMO18 background. CMO18 (also known as H99C) is an attenuated lab passaged derivative of H99S^30^. The H99S *kin1*Δ mutants displayed increased thermosensitivity and enhanced melanin, which is in accordance with the phenotypes observed in the CMO18 *kin1*Δ mutant (Figure S3A and B). The H99S *kin1*Δ mutants also showed decreased capsule production (Supplementary Figure S3C), however, which was in stark contrast to the increased capsule production of the CMO18 *kin1*Δ mutant (Figure 3B). Virulence has been reported to be attenuated in the *kin1*Δ mutant of *C. neoformans*^31^. The *sho1*Δ and *ypk1*Δ mutants both exhibited thermosensitivity and produced enlarged capsules (Supplementary Figure S3A and C). In terms of melanin production, the *sho1*Δ mutant exhibited levels of melanin production comparable to wild-types, but the *ypk1*Δ mutant exhibited highly defective melanin production (Supplementary Figure S2B). To further confirm the role of Sho1 and Ypk1 in the virulence of *C. neoformans*, we performed *in vivo* virulence assays for the *sho1*Δ and *ypk1*Δ mutants using a nasal inhalation-murine cryptococcosis model. The *ypk1*Δ mutant was avirulent (Supplementary Figure S3D), which agrees with a recent report in which it was demonstrated that *ypk1*Δ mutant mice were avirulent in a tail vein-injected murine model of systemic cryptococcosis^32^. In contrast, the *sho1*Δ mutant exhibited normal virulence (Supplementary Figure S3D).

In the tests described above, network-assisted prediction achieved discovery rates of 10% (4 of 40), 11.11% (4 of 36), and 27.03% (10 of 37) for capsule formation, melanin production, and thermotolerance, respectively. Madhani's study previously tested 1,093 of the 1,201 deletion strains for the three virulence phenotypes and reported 16, 40, and 104 validated genes for capsule formation, melanin production, and thermotolerance, respectively (discovery rate of 1.46%, 3.66%, and 9.52%, respectively). Our network-assisted genetic screen, therefore, identified genes for three virulence phenotypes with ~6.8-, ~3-, and ~2.8-fold enrichment over Madhani's screen for capsule formation, melanin production, and thermotolerance genes, respectively (p < 0.01 for all phenotypes based on a binomial test). Notably, 12 of the 29 identified genes (~41%) for the *C. neoformans* virulence phenotypes could not have been predicted by YeastNet-derived links alone, due to the lack of either conserved genes (five of the 12) or conserved links. This result clearly demonstrates the importance of species-specific data for the prediction of *C. neoformans* pathogenicity.

### CryptoNet identifies new antifungal drug resistance genes

One major reason that cryptococcosis treatment fails is antifungal drug resistance. The emerging complexity of the drug-mediated cell death process suggests that a group of drug resistance genes collaborate to overcome drug stress. The elucidation of the complete set of drug resistance genes may therefore facilitate the development of more efficacious antifungal treatments. Some *C. neoformans* genes that change their expression levels during the early adaptation to drug stress may provide clues about the mode of action of antifungal drugs and cellular strategies to overcome drug stress. A majority of signature genes result, however, from indirect effects of target pathway perturbations. The identification of genes that directly contribute to the drug response from gene expression data is hampered, therefore, by confounding signals from indirect effects. Moreover, many cellular processes, including drug resistance, may be regulated by post-transcriptional mechanisms. We therefore require a new search algorithm for drug resistance genes to complement the gene expression-based approach.

Exposing cells to a drug poses a challenge that can trigger expression of many genes that contribute to drug stress resistance. In the functional gene network, such up-regulated genes may be neighbors of the same gene, which is the hub of the network among them. We hypothesized that hub genes connected to many genes that are up-regulated during a drug challenge are likely to be drug resistance genes. To identify such hub genes, we formulated a method called ‘context-associated hub' (see Figure 1 and Supplementary Figure S1). This method requires two gene sets. One set is for the subnetwork, which is composed of a hub gene connected to no less than 50 neighbors by CryptoNet and its neighbors. We predefined 2,135 subnetworks with 2,135 hubs. The other set is a set of genes that are up-regulated during a drug challenge. We used 230 *C. neoformans* genes that exhibited >2-fold up-regulation upon treatment with fluconazole^6^ (Supplementary Table S4). For the given pair of gene sets, one for the 230 up-regulated genes and the other for the neighboring genes from one of the 2,135 subnetworks, we measured the significance of the gene-set association by Fisher's exact test. If the neighbors for a hub gene are significantly enriched among the genes up-regulated by fluconazole, then the corresponding hub gene is considered to be associated with resistance to fluconazole treatment (i.e., a context-associated hub). These algorithms are implemented in the ‘*Find context-associated hub genes*' search option on the CryptoNet web server. Using this search option, we found that 94 of the 2,135 hub genes were significantly associated with a resistance to fluconazole treatment (*p-value* < 0.05). We found that 16 of these 94 candidates genes are known to be involved in the ergosterol pathway (see Supplementary Table S5), which is a known target pathway of fluconazole. This result suggests that the regulation of sterol biosynthesis is a major mechanism of fluconazole resistance. Notably, seven of the 16 (~44%) retrieved ergosterol pathway genes were not up-regulated during fluconazole treatment, which demonstrates that the network-assisted method complements the expression information.

**Figure 5:**
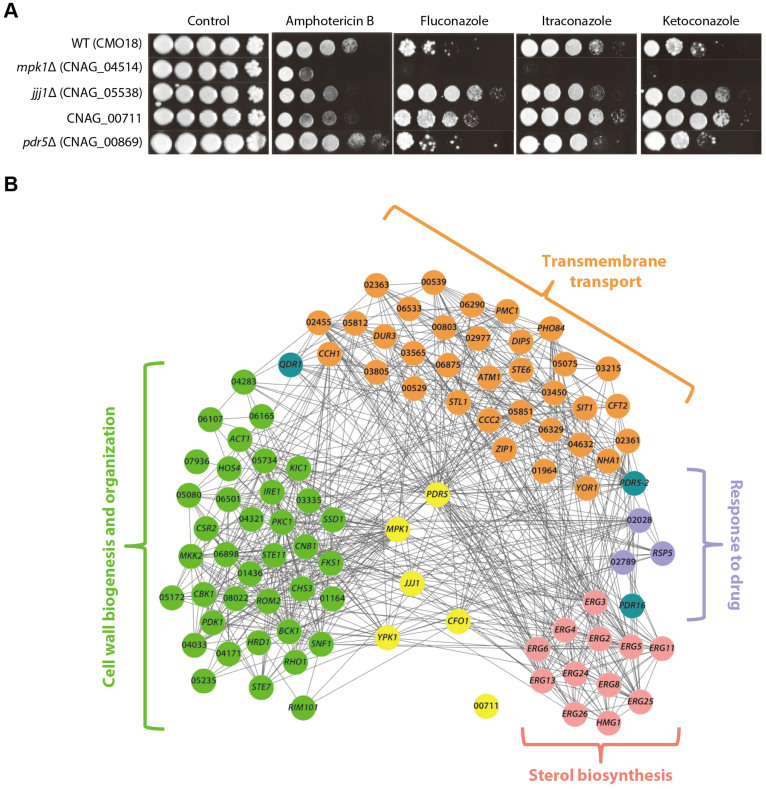
Novel genes for antifungal drug resistance. (A) The antifungal drug resistance test. Cells grown to the mid-logarithmic phase were 10-fold serially diluted (1 to 10^4^) and spotted on YPD medium containing the indicated concentration of azole drugs (fluconazole: 16 μg/ml; itraconazole: 0.06 μg/mL; and ketoconazole: 0.25 μg/mL) or amphotericin B (1.2 μg/mL), and further incubated at 30°C for the antifungal drug resistance assay. (B) The neighbors of six novel drug resistance genes are enriched for four biological processes: sterol biosynthetic process, response to drug, transmembrane transport, and cell wall organization and biogenesis. The genes for the four enriched processes show high modularity in CryptoNet.

We tested 11 candidate genes for fluconazole resistance that have available mutant strains in the Madhani collection (see Supplementary Table S5). We also tested the same candidate genes for other azole drugs, including itraconazole and ketoconazole, which also inhibit lanosterol 14α-demethylase, an enzyme that is required for the conversion of lanosterol to ergosterol. We also tested amphotericin B, which belongs to the polyene antifungal agents that change the permeability of the fungal membrane by binding to ergosterol, which in turn leads to cell death^33^. Four of the 11 tested genes, CNAG_04514 (*MPK1*), CNAG_05538 (*JJJ1*), CNAG_00711, and CNAG_00869 (*PDR5*), exhibited an increased resistance or sensitivity to the azole drugs and amphotericin B compared with the wild-type strain (discovery rate = 36%, Figure 5A). Three genes, *JJJ1*, *PDR5*, and CNAG_00711, previously have not been reported to be involved in antifungal drug resistance. The deletion of *MPK1*, which is known to regulate the integrity of the cell wall in response to the antifungal drugs nikkomycin Z, caspofungin, and FK506^34^, resulted in severe sensitivity to azoles and amphotericin B. A significant increase in sensitivity to fluconazole in the *MPK1*-deleted study also was observed in a previous study^32^. Two additional drug resistance genes that have been reported previously were included among our 94 candidate genes: *YPK1*, the deletion of which significantly increases sensitivity to fluconazole^32^, and CNAG_06241 (*CFO1*), the deletion of which increases sensitivity to both amphotericin B and fluconazole^35^.

CryptoNet can also provide insights into the pathways that underlie drug resistance. The validated drug resistance genes may be connected to other genes in relevant pathways. We therefore measured the enrichment of pathway annotations related to drug resistance among their network neighbors. Given that the majority of the *C. neoformans* genes are not yet annotated into pathways, we employed the Gene Ontology biological process (GOBP) annotations for the *S. cerevisiae* orthologs. We found that four GOBP terms relevant to drug response or ergosterol biosynthesis were significantly enriched: sterol biosynthetic process (GO:0016126), response to drug (GO:0042493), transmembrane transport (GO:0055085), and cell wall organization and biogenesis (GO:0071554) (*p-*value < 10^−4^, Fisher's exact test). CryptoNet reveals a modular organization of the pathways to which the drug resistance genes are highly connected (Figure 5B). Notably, CNAG_00711 is connected to neither the four relevant pathway genes nor other drug resistance genes. To infer pathway functions for this uncharacterized gene, we examined enriched GOBP terms among its network neighbors using the search option ‘*Infer functions from network neighbors*' (see Supplementary Figure S1) from the CryptoNet web server (Supplementary Table S6). The top two predicted GOBP terms for CNAG_00711 are NADH oxidation (GO:0006116), which is related to the process of delivering electrons to the electron transport chain in the mitochondria, and intracellular accumulation of glycerol (GO:0006973) rendered by *GPD1* (glycerol 3-phosphate dehydrogenase, GPDH). Previous studies have shown that mitochondrial dysfunction related to energy generation is required for azole susceptibility in *C. albicans*^36^, and expression of Gpd3 (a putative GPDH protein) is up-regulated 25-fold in an azole-resistant *C. glabrata* strain^37^. These findings suggest that CNAG_00711 modulates azole drug resistance via either energy production or glycerol synthesis mediated by Gpd1. Further studies are needed to elucidate the mechanisms of azole drug resistance that are connected to CNAG_00711. Taken together, we conclude that CryptoNet provides new insights into drug resistance in *C. neoformans*.

## Discussion

In this study, we have demonstrated the feasibility of network-assisted identification of novel genes for pathogenicity and antifungal drug resistance in *C. neoformans*. Network-assisted gene prioritization requires two technical components. First is the need to construct a highly accurate and comprehensive gene network for the target species. We have demonstrated that heterogeneous genomics data can be effectively integrated into a single gene network for *C. neoformans*. CryptoNet is distinct from the previously constructed fungal networks from several clinical and evolutionary perspectives. CryptoNet is the first genome-scale co-functional network for a fungal pathogen and covers ~81% of the coding genome. In our study, we demonstrated the power of CryptoNet in the study of pathogenicity and drug resistance, which are major challenges in the development of medicine for infectious diseases. CryptoNet is also the first genome-scale co-functional network for a fungal species in the basidiomycota phylum, as *S. cerevisiae* belongs to the ascomycota phylum. Given the evolutionary distance between *C. neoformans* and *S. cerevisiae*, the orthology-based network from YeastNet makes a limited contribution to the *C. neoformans* gene network (e.g., Figure 2C).

The second key technical component for network-assisted gene prioritization is the need to develop network algorithms that prioritize genes for the phenotype of interest. Here, we used two distinct network algorithms for gene prioritization: ‘guilt-by-association' and ‘context-associated hub'. We successfully identified 29 virulence genes using guilt-by-association by propagating information from 73 known virulence genes in CryptoNet. Several known and novel virulence genes were confirmed and discovered in this study, respectively, and we provide an in-depth discussion about their molecular functions in Supplementary Table S7. For the prediction of antifungal resistance genes, we used an alternative network approach, context-associated hub, which employs expression information that depicts the given cellular context in combination with CryptoNet, to identify six drug resistance genes, including 3 novel genes. Taken together, these results demonstrate the versatility of CryptoNet with other types of biological information incorporated.

There are some potential limitations to the use of CryptoNet and network-assisted predictive genetics. First, we can only make predictions for genes included in CryptoNet, which currently covers ~81% of the coding genes. Second, CryptoNet cannot be utilized to determine the causality of functional relationships between genes. Third, network-based inferences require known pathway genes to apply the guilt-by-association method or expression data to use the context-associated hub method. The limitation of network coverage will be overcome gradually as more genomics or proteomics data for *C. neoformans* becomes available. For example, large-scale protein-protein interaction data for *C. neoformans* will significantly expand the current network view.

From the systems genetics perspective, in which we imagine each phenotype as a system composed of genetic components, the gene-to-phenotype association mapping is critical to the understanding of the genetic organization of complex phenotypes. A bottom-up reconstruction of phenotypic systems as gene networks will not only account for the emergent properties of genetic perturbations but also provide novel functional insights into the individual genetic components of the relevant pathways. The integration of large amounts of high-throughput data produces a genome-scale gene network, from which a list of highly probable candidate genes can be generated to increase the rate of discovery. As an intermediary between high-throughput and candidate gene approaches, gene networks therefore can accelerate our progress in understanding the genetics of multiple complex phenotypes in fungal pathogens, such as pathogenesis and drug resistance. Because every component of our proposed method can be adopted for any given species, this method is applicable to the study of other pathogenic and saprobic microbes.

## Methods

### Sequences and functional annotation data for *Cryptococcus neoformans*

The templates for the genome sequence and functional annotations employed in this study are *C. neoformans* var. *grubii* H99 (serotype A) and *S. cerevisiae* GO annotations, which are described in the Supplementary Methods.

### Benchmarking and integration of co-functional links (Bayesian data integration)

Network benchmarking and data integration using Bayesian data integration were performed as previously described^13^. For more details, see the Supplementary Methods.

### Construction of *ypk1*Δ, *sho1*Δ and *kin1*Δ mutants

The selected genes [CNAG_01938 (*KIN1*), CNAG_04678 (*YPK1*), or CNAG_05499 (Sc*SHO1*)] were deleted in the H99S serotype A strain. For more details, see the Supplementary Methods.

### Assay for virulence factor production, thermotolerance, and antifungal drug resistance

*C. neoformans* growth conditions, capsule assays, and melanin assays were performed as previously described^38^. Further information can be found in the Supplementary Methods. The antifungal drug assay and thermotolerance tests also were performed as previously described^39^.

### *Galleria mellonella* infection assay and *in vivo* mouse study

The *G. mellonella* infection assay was performed by following the previously described methods^40^ with minor modifications, the details of which can be found in the Supplementary Methods. For the *in vivo* mouse study, we used four- to six-week-old female A/Jcr mice. The experiment was performed as previously described^41^ with minor modifications, the details of which can be found in the Supplementary Methods.

### A web-based prediction server for *C. neoformans* biology

All network-assisted predictions for *C. neoformans* genes described in this study can be performed using the public web server at www.inetbio.org/cryptonet.

## Author Contributions

H.K., K.J., Y.B. and I.L. conceived the project and wrote the manuscript. H.K. constructed and analyzed the network model. K.J. performed the experiments with the assistance of T.K. and S.M. Y.-L.C. performed the animal studies. J.H. supervised the animal studies. J.S., J.E.S. and S.H. contributed to the data analysis pipeline developments. G.J. constructed the coding genome for *Cryptococcus neoformans* var. *grubii* (serotype A). Y.B. supervised the experimental analysis. I.L. supervised the modeling and bioinformatics analysis. H.K., K.J., Y.B., J.H. and I.L. edited the manuscript.

## Supplementary Material

Supplementary Information Supplementary Materials and Methods

## References

[b1] Hoang L. M., Maguire J. A., Doyle P., Fyfe M. & Roscoe D. L. *Cryptococcus neoformans* infections at Vancouver Hospital and Health Sciences Centre (1997–2002): epidemiology, microbiology and histopathology. J. Med. Microbiol. 53, 935–940 (2004). 15314203 10.1099/jmm.0.05427-0

[b2] Park B. J. *et al.* Estimation of the current global burden of cryptococcal meningitis among persons living with HIV/AIDS. AIDS 23, 525–530 (2009). 19182676 10.1097/QAD.0b013e328322ffac

[b3] Kwon-Chung K. J. & Rhodes J. C. Encapsulation and melanin formation as indicators of virulence in *Cryptococcus neoformans*. Infect. Immun. 51, 218–223 (1986). 3079732 10.1128/iai.51.1.218-223.1986 PMC261090

[b4] Wang Y., Aisen P. & Casadevall A. *Cryptococcus neoformans* melanin and virulence: mechanism of action. Infect. Immun. 63, 3131–3136 (1995). 7622240 10.1128/iai.63.8.3131-3136.1995 PMC173427

[b5] Pfaller M. A. Antifungal drug resistance: mechanisms, epidemiology and consequences for treatment. Am. J. Med. 125, S3–13 (2012). 22196207 10.1016/j.amjmed.2011.11.001

[b6] Florio A. R. *et al.* Genome-wide expression profiling of the response to short-term exposure to fluconazole in *Cryptococcus neoformans* serotype A. BMC Microbiol. 11, 97 (2011). 21569340 10.1186/1471-2180-11-97 PMC3119188

[b7] Kim M. S. *et al.* Comparative transcriptome analysis of the CO_2_ sensing pathway via differential expression of carbonic anhydrase in *Cryptococcus neoformans*. Genetics 185, 1207–1219 (2010). 20516494 10.1534/genetics.110.118315 PMC2927750

[b8] Maeng S. *et al.* Comparative transcriptome analysis reveals novel roles of the Ras and cyclic AMP signaling pathways in environmental stress response and antifungal drug sensitivity in *Cryptococcus neoformans*. Eukaryot. Cell 9, 360–378 (2010). 20097740 10.1128/EC.00309-09 PMC2837985

[b9] Liu O. W. *et al.* Systematic genetic analysis of virulence in the human fungal pathogen *Cryptococcus neoformans*. Cell 135, 174–188 (2008). 18854164 10.1016/j.cell.2008.07.046 PMC2628477

[b10] Ideker T. & Sharan R. Protein networks in disease. Genome Res. 18, 644–652 (2008). 18381899 10.1101/gr.071852.107 PMC3863981

[b11] Lee I. Network approaches to the genetic dissection of phenotypes in animals and humans. Anim. Cells Syst. 17, 75–79 (2013).

[b12] Lehner B. & Lee I. Network-guided genetic screening: building, testing and using gene networks to predict gene function. Brief. Funct. Genomic. Proteomic. 7, 217–227 (2008). 18445637 10.1093/bfgp/eln020

[b13] Kim H. *et al.* YeastNet v3: a public database of data-specific and integrated functional gene networks for *Saccharomyces cerevisiae*. Nucleic Acids Res. 42, D731–736 (2014). 24165882 10.1093/nar/gkt981 PMC3965021

[b14] McGary K. L. *et al.* Systematic discovery of nonobvious human disease models through orthologous phenotypes. Proc. Natl. Acad. Sci. U. S. A. 107, 6544–6549 (2010). 20308572 10.1073/pnas.0910200107 PMC2851946

[b15] Haynes B. C. *et al.* Toward an integrated model of capsule regulation in *Cryptococcus neoformans*. PLoS Pathog. 7, e1002411 (2011). 22174677 10.1371/journal.ppat.1002411 PMC3234223

[b16] Kozubowski L., Thompson J. W., Cardenas M. E., Moseley M. A. & Heitman J. Association of calcineurin with the COPI protein Sec28 and the COPII protein Sec13 revealed by quantitative proteomics. PLoS One 6, e25280 (2011). 21984910 10.1371/journal.pone.0025280 PMC3184950

[b17] Pukkila-Worley R. *et al.* Transcriptional network of multiple capsule and melanin genes governed by the *Cryptococcus neoformans* cyclic AMP cascade. Eukaryot. Cell 4, 190–201 (2005). 15643074 10.1128/EC.4.1.190-201.2005 PMC544166

[b18] Linde J. *et al.* Regulatory interactions for iron homeostasis in *Aspergillus fumigatus* inferred by a Systems Biology approach. BMC Syst. Biol. 6, 6 (2012). 22260221 10.1186/1752-0509-6-6 PMC3305660

[b19] Linde J., Wilson D., Hube B. & Guthke R. Regulatory network modelling of iron acquisition by a fungal pathogen in contact with epithelial cells. BMC Syst. Biol. 4, 148 (2010). 21050438 10.1186/1752-0509-4-148 PMC3225834

[b20] Altwasser R., Linde J., Buyko E., Hahn U. & Guthke R. Genome-wide scale-free network inference for *Candida albicans*. Front. Microbiol. 3, 51 (2012). 22355294 10.3389/fmicb.2012.00051 PMC3280432

[b21] Kim E., Kim H. & Lee I. JiffyNet: a web-based instant protein network modeler for newly sequenced species. Nucleic Acids Res. 41, W192–197 (2013). 23685435 10.1093/nar/gkt419 PMC3692116

[b22] Yu H. *et al.* Annotation transfer between genomes: protein-protein interologs and protein-DNA regulogs. Genome Res. 14, 1107–1118 (2004). 15173116 10.1101/gr.1774904 PMC419789

[b23] Kanehisa M., Goto S., Sato Y., Furumichi M. & Tanabe M. KEGG for integration and interpretation of large-scale molecular data sets. Nucleic Acids Res. 40, D109–114 (2012). 22080510 10.1093/nar/gkr988 PMC3245020

[b24] Lee I., Blom U. M., Wang P. I., Shim J. E. & Marcotte E. M. Prioritizing candidate disease genes by network-based boosting of genome-wide association data. Genome Res. 21, 1109–1121 (2011). 21536720 10.1101/gr.118992.110 PMC3129253

[b25] Dimmer E. C. *et al.* The UniProt-GO Annotation database in 2011. Nucleic acids research 40, D565–570 (2012). 22123736 10.1093/nar/gkr1048 PMC3245010

[b26] Cramer K. L., Gerrald Q. D., Nichols C. B., Price M. S. & Alspaugh J. A. Transcription factor Nrg1 mediates capsule formation, stress response and pathogenesis in *Cryptococcus neoformans*. Eukaryot. Cell 5, 1147–1156 (2006). 16835458 10.1128/EC.00145-06 PMC1489281

[b27] Hu G., Cheng P. Y., Sham A., Perfect J. R. & Kronstad J. W. Metabolic adaptation in *Cryptococcus neoformans* during early murine pulmonary infection. Mol. Microbiol. 69, 1456–1475 (2008). 18673460 10.1111/j.1365-2958.2008.06374.x PMC2730461

[b28] Wang Y., Liu T. B., Patel S., Jiang L. & Xue C. The casein kinase I protein Cck1 regulates multiple signaling pathways and is essential for cell integrity and fungal virulence in *Cryptococcus neoformans*. Eukaryot. Cell 10, 1455–1464 (2011). 21926330 10.1128/EC.05207-11 PMC3209051

[b29] Yang J. *et al.* Regulation of virulence factors, carbon utilization and virulence by *SNF1* in *Cryptococcus neoformans* JEC21 and divergent actions of SNF1 between cryptococcal strains. Fungal Genet. Biol. 47, 994–1000 (2010). 20719250 10.1016/j.fgb.2010.08.002

[b30] Janbon G. *et al.* Analysis of the genome and transcriptome of *Cryptococcus neoformans* var. *grubii* reveals complex RNA expression and microevolution leading to virulence attenuation. PLoS Genet. 10, e1004261 (2014). 24743168 10.1371/journal.pgen.1004261 PMC3990503

[b31] Mylonakis E. *et al.* *Cryptococcus neoformans* Kin1 protein kinase homologue, identified through a *Caenorhabditis elegans* screen, promotes virulence in mammals. Mol. Microbiol. 54, 407–419 (2004). 15469513 10.1111/j.1365-2958.2004.04310.x

[b32] Lee H., Khanal Lamichhane A., Garraffo H. M., Kwon-Chung K. J. & Chang Y. C. Involvement of PDK1, PKC and TOR signalling pathways in basal fluconazole tolerance in *Cryptococcus neoformans*. Mol. Microbiol. 84, 130–146 (2012). 22339665 10.1111/j.1365-2958.2012.08016.x PMC3313003

[b33] Sanglard D., Coste A. & Ferrari S. Antifungal drug resistance mechanisms in fungal pathogens from the perspective of transcriptional gene regulation. FEMS Yeast Res. 9, 1029–1050 (2009). 19799636 10.1111/j.1567-1364.2009.00578.x

[b34] Kraus P. R., Fox D. S., Cox G. M. & Heitman J. The *Cryptococcus neoformans* MAP kinase Mpk1 regulates cell integrity in response to antifungal drugs and loss of calcineurin function. Mol. Microbiol. 48, 1377–1387 (2003). 12787363 10.1046/j.1365-2958.2003.03508.x PMC1635492

[b35] Jung W. H., Hu G., Kuo W. & Kronstad J. W. Role of ferroxidases in iron uptake and virulence of *Cryptococcus neoformans*. Eukaryot. Cell 8, 1511–1520 (2009). 19700638 10.1128/EC.00166-09 PMC2756870

[b36] Sun N. *et al.* Azole susceptibility and transcriptome profiling in *Candida albicans* mitochondrial electron transport chain complex I mutants. Antimicrob. Agents Chemother. 57, 532–542 (2013). 23147730 10.1128/AAC.01520-12 PMC3535965

[b37] Rogers P. D., Vermitsky J. P., Edlind T. D. & Hilliard G. M. Proteomic analysis of experimentally induced azole resistance in *Candida glabrata*. J. Antimicrob. Chemother. 58, 434–438 (2006). 16735426 10.1093/jac/dkl221

[b38] Bahn Y. S., Hicks J. K., Giles S. S., Cox G. M. & Heitman J. Adenylyl cyclase-associated protein Aca1 regulates virulence and differentiation of *Cryptococcus neoformans* via the cyclic AMP-protein kinase A cascade. Eukaryot. Cell 3, 1476–1491 (2004). 15590822 10.1128/EC.3.6.1476-1491.2004 PMC539029

[b39] Ko Y. J. *et al.* Remodeling of global transcription patterns of *Cryptococcus neoformans* genes mediated by the stress-activated HOG signaling pathways. Eukaryot. Cell 8, 1197–1217 (2009). 19542307 10.1128/EC.00120-09 PMC2725552

[b40] Mylonakis E. *et al.* *Galleria mellonella* as a model system to study *Cryptococcus neoformans* pathogenesis. Infect. Immun. 73, 3842–3850 (2005). 15972469 10.1128/IAI.73.7.3842-3850.2005 PMC1168598

[b41] Cheon S. A. *et al.* Unique evolution of the UPR pathway with a novel bZIP transcription factor, Hxl1, for controlling pathogenicity of *Cryptococcus neoformans*. PLoS Pathog. 7, e1002177 (2011). 21852949 10.1371/journal.ppat.1002177 PMC3154848

