# Supplementary material for: Network-assisted genetic dissection of pathogenicity and drug resistance in the opportunistic human pathogenic fungus Cryptococcus neoformans
Source: Sci Rep. 2015 Mar 5;5:8767. doi: 10.1038/srep08767 (PMC4350084; doi:10.1038/srep08767)
Supplement: Supplementary Information — Supplementary Materials and Methods [file srep08767-s1.pdf]

Supplementary Materials and Methods

Network-assisted genetic dissection of pathogenicity and drug resistance in the opportunistic human pathogenic fungus *Cryptococcus neoformans*

Hanhae Kim, Kwang-Woo Jung, Shinae Maeng, Ying-Lien Chen, Junha Shin, Jung Eun Shim, Sohyun Hwang, Guilhem Janbon, Taeyup Kim, Joseph Heitman, Yong-Sun Bahn, and Insuk Lee

☐ **Contents**

- **Supplementary Figure S1**
- **Supplementary Figure S2**
- **Supplementary Figure S3**
- **Supplementary Table S1**
- **Supplementary Table S2**
- **Supplementary Table S3**
- **Supplementary Table S4**
- **Supplementary Table S5**
- **Supplementary Table S6**
- **Supplementary Table S7**
- **Supplementary Table S8**
- **Supplementary Table S9**
- **Supplementary Methods**
- **Supplemental References**

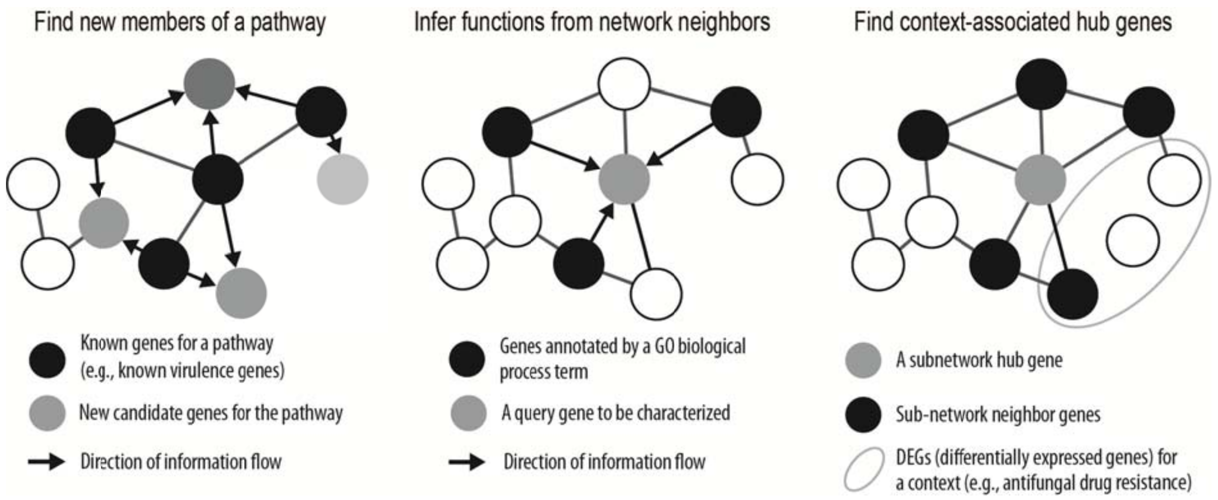

Supplementary Figure S1. A schematic summary of network search options for hypothesis generation using CryptoNet (A) 'Find new members of a pathway' search option is used to prioritize genes for pathways or phenotypes (gray node) such as virulence by propagating information of known genes (black node) through the network. Candidate genes are prioritized by sum of edge scores to known genes. Here, darker gray nodes represent more likely candidate genes. (B) 'Infer functions from network neighbors' search option is used to prioritize functions for a query gene (gray node). GO biological process annotations among neighbor genes are collected and algorithm rank them from the most enriched one. Here, an annotation term is most enriched among genes marked by black color. (C) 'Find context associated hub genes' search option is used to identify a sub-network hub gene (gray node) associated with a context of interest. This option is feasible to calculate significant overlap between submitted differentially expressed genes (DEGs, nodes in the larger circle) and neighbor genes of the sub-network hub gene (black node) by Fisher's exact test. If a sub-network hub gene significantly possesses DEGs as its neighbor genes, the sub-network hub gene is defined as a context associated hug gene for the given context.

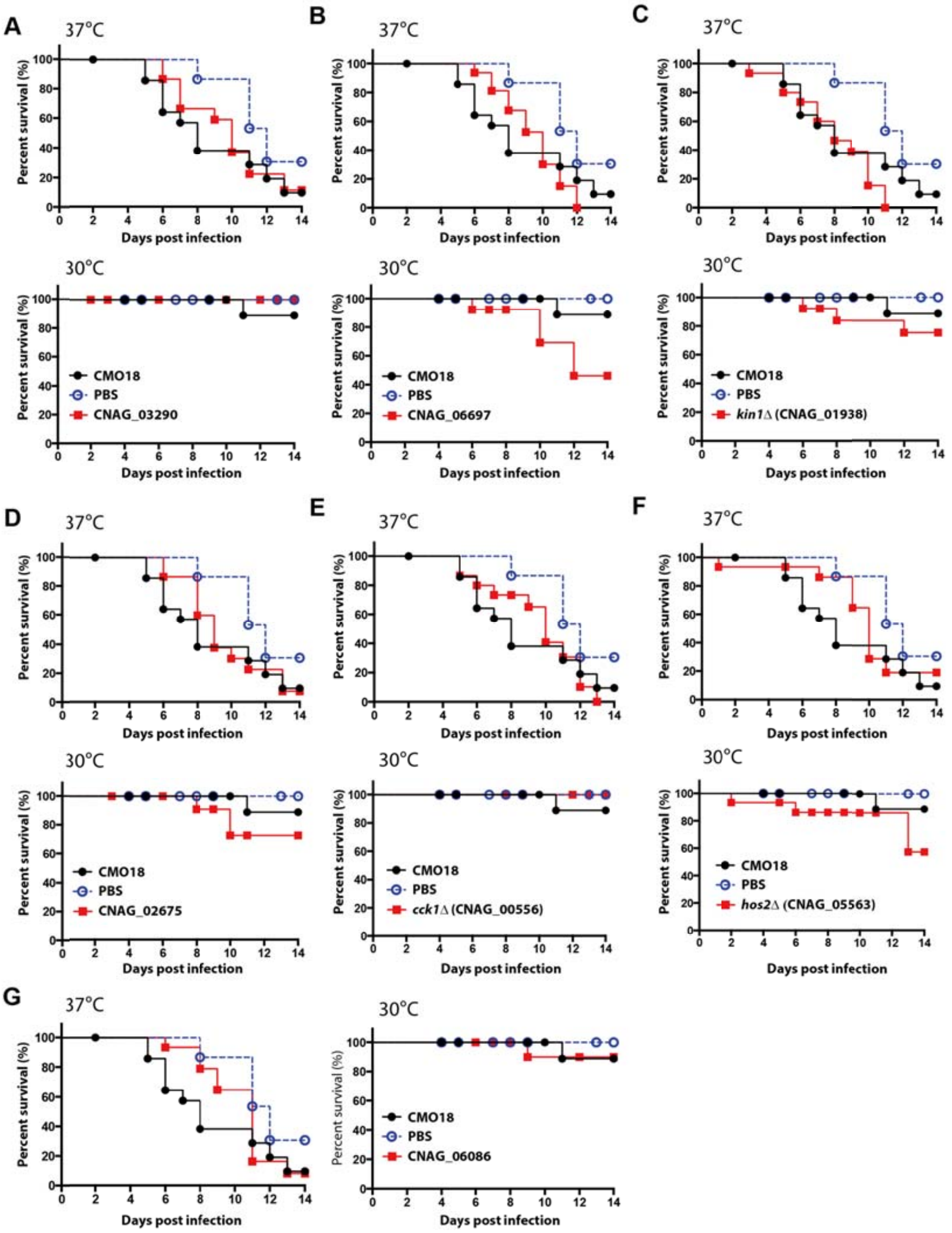

Supplementary Figure S2. ***in vivo* virulence assay of candidate genes by CryptoNet using a wax moth model system** Each larva was infected with 800,000 *Cryptococcus* cells and incubated at 30°C or 37°C. Percent survival (%) was monitored for 2 weeks post-infection. Percent survival (%) was monitored for 2 weeks post-infection. *P*=0.3755 and *P*=0.3458 for CMO18 vs. CNAG_03290 at 37°C or 30°C, *P*=0.2206 and *P*=0.0772 for CMO18 vs. CNAG_06697 at 37°C or 30°C, *P*=0.7671 and *P*=0.3040 for CMO18 vs. *kin1*Δ (CNAG_01938) mutant at 37°C or 30°C, *P*=0.2159 and *P*=0.2033 for CMO18 vs. CNAG_02675 at 37°C or 30°C, *P*=0.4473 and *P*=0.3173 for CMO18 vs. *cck1*Δ (CNAG_00556) mutant at 37°C or 30°C, *P*=0.2113 and *P*=0.1461 for CMO18 vs. *hos2*Δ (CNAG_05563) mutant at 37°C or 30°C, *P*=0.0905 and *P*=0.5858 for CMO18 vs. CNAG_06086 at 37°C or 30°C, respectively.

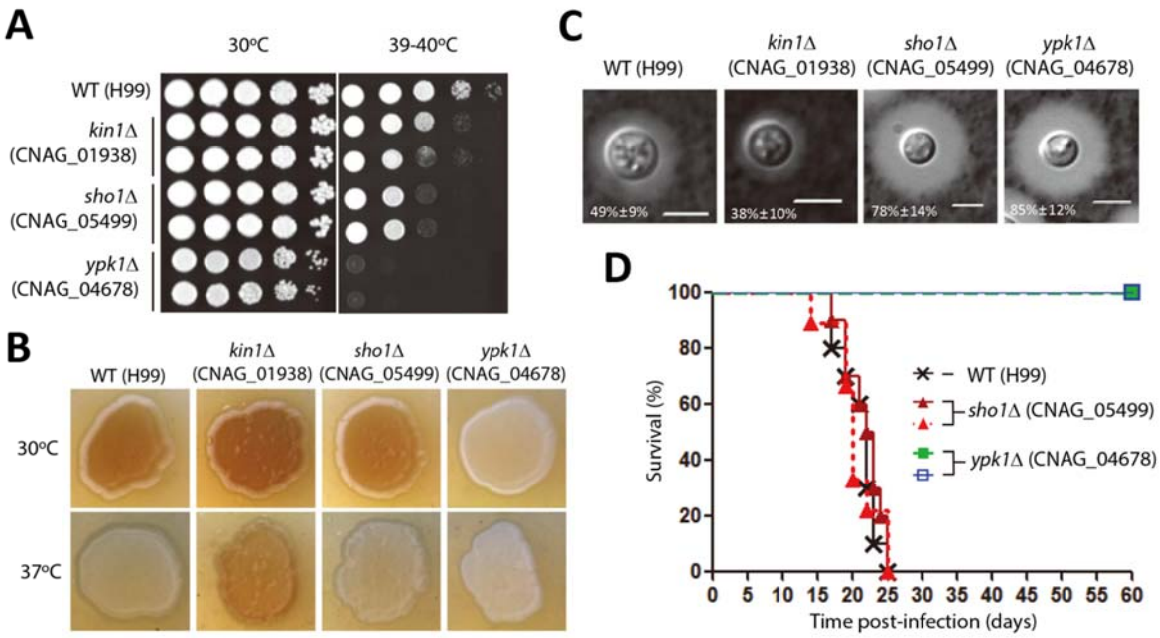

Supplementary Figure S3. **Experimental validations for Kin1, Ypk1, and Sho1 that were predicted by CryptoNet to be involved in virulence regulation in *C. neoformans*** (A) For thermotolerance test, each strain grown overnight was 10-fold serially diluted (1 to 10^4), spotted on YPD medium, and further incubated at 30°C, or 39°C for 2-4 days. (B) Each *C. neoformans* strain was spotted on Niger seed medium containing the indicated concentration of glucose (0.5%) and incubated at 30°C or 37°C. The images were photographed by using a microscope (Motic Microscope) equipped with a digital camera (Pro-Microscan No. 5888). (C) For capsule production measurement, each strain was spotted and cultured on DME agar medium at 30°C for 2 days. Capsules were visualized by India ink staining. Relative capsule diameter was described in the picture. The scale bar represents 10 µm. The images were photographed by using a SPOT insight digital camera (Diagnostic Instrument Inc.) (D) A/Jcr mice were infected with 10^5 cells of WT and two independent mutants by intranasal instillation. Survival (%) was monitored daily for 60 days after infection. Strains were: WT (H99), *kin1*Δ mutants (YSB1716 and YSB1718), *sho1*Δ mutants (YSB1719 and YSB1720) and *ypk1*Δmutants (YSB1735 and YSB1736).

Supplementary Table S1. Fourteen distinct data types incorporated into the CryptoNet

Evidence code	Evidence description	# unique genes	# unique gene pairs
CN-CC	Inferred links by co-citation of two genes across full texts of 609 PubMed central (PMC) articles for *C. neoformans* biology	1066	23217
CN-CX	Inferred links by co-expression pattern of two *C. neoformans* genes across gene expression profiles obtained from GEO database	5699	288517
CN-DC	Inferred links by co-occurrence of protein domains between two *C. neoformans* coding genes	2844	7437
CN-GN	Inferred links by similar genomic context of bacterial orthologs of two *C. neoformans* genes	2386	36495
CN-PG	Inferred links by similar phylogenetic profiles between two *C. neoformans* genes	1458	36514
HS-HT	Associalogs of high-throughput human protein-protein interactions	1079	5379
HS-LC	Associalogs of small/medium-scale human protein-protein interactions (collected from protein-protein interaction data bases)	1473	14619
SC-CC	Associalogs of yeast gene links by co-citation of two yeast genes across 46,111 PubMed Medline articles for yeast biology	2254	28539
SC-CX	Associalogs of yeast gene links by co-expression pattern	2613	170489
SC-DC	Associalogs of yeast gene links by co-occurrence of protein domains between two yeast coding genes	1561	6731
SC-GT	Associalogs of yeast gene links by similar profiles of yeast genetic interaction context	1960	4089
SC-HT	Associalogs of high-throughput yeast protein-protein interactions	2543	64617
SC-LC	Associalogs of small/medium-scale yeast protein-protein interactions (collected from protein-protein interaction data bases)	2527	25928
SC-TS	Associalogs of protein interactions from yeast tertiary structures of complexes	593	1395
CryptoNet	A integrated gene network for *C. neoformans*	5649	156506

Supplementary Table S2. 73 Cryptococcus neoformans genes for three different virulence phenotypes collected from literature

Category	H99 locus ID	Gene name	Reference
Capsule	CNAG_00769	*PBS2*	Mol Biol Cell. 2005 May;16(5):2285-300.
Capsule	CNAG_03202	*CAC1*	Eukaryot Cell. 2002 Feb;1(1):75-84.
Capsule	CNAG_03818	*SSK1*	Mol Biol Cell. 2006 Jul;17(7):3122-35.
Capsule	CNAG_00570	*PKR1*	Mol Cell Biol. 2001 May;21(9):3179-91.
Capsule	CNAG_05431	*RIM101*	PLoS Pathog. 2010 Feb 19;6(2):e1000776.
Capsule	CNAG_01626	*ADA2*	PLoS Pathog. 2011 Dec;7(12):e1002411
Capsule	CNAG_02885	*CAP64*	Infect Immun. 1996 Jun;64(6):1977-83
Capsule	CNAG_00721	*CAP59*	Mol Cell Biol. 1994 Jul;14(7):4912-9
Capsule	CNAG_04090	*ATF1*	Genetics. 2010 Aug;185(4):1207-19.
Capsule	CNAG_05063	*SSK2*	Eukaryot Cell. 2007 Dec;6(12):2278-89.
Capsule	CNAG_04864	*CIR1*	PLoS Biol. 2006 Nov;4(12):e410.
Capsule	CNAG_01845	*PKC1*	Eukaryot Cell. 2008 Oct;7(10):1685-98.
Capsule	CNAG_04505	*GPA1*	Eukaryot Cell. 2004 Dec;3(6):1476-91.
Capsule	CNAG_05465	*GIB2*	J Biol Chem. 2006 Oct 27;281(43):32596-605.
Capsule	CNAG_06301	*SCH9*	Curr Genet. 2004 Nov;46(5):247-55.
Capsule	CNAG_05817	*GMT1*	Eukaryot Cell. 2007 May;6(5):776-85.
Capsule	CNAG_03670	*IRE1*	PLoS Pathog. 2011 Aug;7(8):e1002177
Capsule	CNAG_07554	*CAP10*	J Bacteriol. 1999 Sep;181(18):5636-43.
Capsule	CNAG_02702	*CLC-A*	Mol Microbiol. 2003 Nov;50(4):1271-81.
Capsule	CNAG_05218	*ACA1*	Eukaryot Cell. 2004 Dec;3(6):1476-91
Capsule	CNAG_00600	*CAP60*	Infect Immun. 1998 May;66(5):2230-6
Capsule	CNAG_01523	*HOG1*	Mol Biol Cell. 2005 May;16(5):2285-300.
Capsule	CNAG_04969	*UGD1*	Eukaryot Cell. 2004 Dec;3(6):1601-8.
Capsule	CNAG_02008	*OVA1*	PLoS Pathog. 2007 Mar;3(3):e42.
Capsule	CNAG_07470	*PDE2*	Eukaryot Cell, Dec. 2005, 4(12) 1971–1981
Capsule	CNAG_01106	*VPH1*	Mol Microbiol. 2001 Nov;42(4):1121-31.
Capsule	CNAG_05081	*PDE1*	Eukaryot Cell, Dec. 2005, 4(12) 1971–1981
Capsule	CNAG_00396	*PKA1*	Mol Cell Biol. 2001 May;21(9):3179-91

Melanin	CNAG_06552	*SNF1*	Fungal Genet Biol. 2010 Dec; 47(12):994-1000.
Melanin	CNAG_00996	*PMT4*	PLoS One. 2009 Jul 27;4(7):e6321.
Melanin	CNAG_00815	*SIT1*	Microbiology. 2007 Jan;153(Pt 1):29-41.
Melanin	CNAG_04215	*MET3*	Microbiology. 2002 Aug;148(Pt 8):2617-25.
Melanin	CNAG_00130	*HRK1*	PLoS ONE 6(4): e18769
Melanin	CNAG_00769	*PBS2*	Mol Biol Cell. 2005 May;16(5):2285-300.
Melanin	CNAG_03409	*SKN7*	Mol Biol Cell. 2006 Jul;17(7):3122-35.
Melanin	CNAG_05218	*ACA1*	Eukaryot Cell. 2004 Dec;3(6):1476-91
Melanin	CNAG_04090	*ATF1*	Genetics. 2010 Aug;185(4):1207-19.
Melanin	CNAG_01523	*HOG1*	Mol Biol Cell. 2005 May;16(5):2285-300.
Melanin	CNAG_02357	*MKK2*	Mol Microbiol. 2005 Oct;58(2):393-408.
Melanin	CNAG_05081	*PDE1*	Eukaryot Cell, Dec. 2005, 4(12) 1971–1981
Melanin	CNAG_04505	*GPA1*	Eukaryot Cell. 2004 Dec;3(6):1476-91.
Melanin	CNAG_03202	*CAC1*	Eukaryot Cell. 2002 Feb;1(1):75-84.
Melanin	CNAG_02867	*PLC1*	Mol Microbiol. 2008 Aug;69(4):809-26.
Melanin	CNAG_03818	*SSK1*	Mol Biol Cell. 2006 Jul;17(7):3122-35.
Melanin	CNAG_07470	*PDE2*	Eukaryot Cell, Dec. 2005, 4(12) 1971–1981
Melanin	CNAG_00396	*PKA1*	Mol Cell Biol. 2001 May;21(9):3179-91
Melanin	CNAG_04864	*CIR1*	PLoS Biol. 2006 Nov;4(12):e410.
Melanin	CNAG_01845	*PKC1*	Mol Microbiol. 2005 Oct;58(2):393-408.
Melanin	CNAG_03465	*LAC1*	J. Exp. Med. 1996 184:377–386.
Melanin	CNAG_03464	*LAC2*	Eukaryot Cell 2005 4(1) 190-201
Melanin	CNAG_05063	*SSK2*	Eukaryot Cell. 2007 Dec;6(12):2278-89.
Thermotolerance	CNAG_02357	*MKK2*	Mol Microbiol. 2005 Oct;58(2):393-408.
Thermotolerance	CNAG_01523	*HOG1*	Mol Biol Cell. 2005 May;16(5):2285-300.
Thermotolerance	CNAG_01845	*PCK1*	Mol Microbiol. 2005 Oct;58(2):393-408.
Thermotolerance	CNAG_01626	*ADA2*	PLoS Pathog. 2011 Dec;7(12):e1002411
Thermotolerance	CNAG_00375	*GCN5*	Eukaryot Cell. 2010 Aug;9(8):1193-202.
Thermotolerance	CNAG_06134	*HXL1*	PLoS Pathog. 2011 Aug;7(8):e1002177
Thermotolerance	CNAG_05348	*CDC42*	Mol Microbiol. 2010 Feb;75(3):763-80
Thermotolerance	CNAG_04388	*SOD2*	Eukaryot Cell. 2005 Jan;4(1):46-54.

Thermotolerance	CNAG_03818	*SSK1*	Mol Biol Cell. 2006 Jul;17(7):3122-35.
Thermotolerance	CNAG_06301	*SCH9*	Curr Genet 2004.Nov 46(5):247-255
Thermotolerance	CNAG_01530	*MGA2*	Eukaryot Cell. 2004 Oct;3(5):1249-60.
Thermotolerance	CNAG_04864	*CIR1*	PLoS Biol. 2006 Nov;4(12):e410.
Thermotolerance	CNAG_04969	*UGD1*	J Biol Chem. 2004 Dec 3;279(49):51669-76.
Thermotolerance	CNAG_04755	*BCK1*	Mol Microbiol. 2005 Oct;58(2):393-408.
Thermotolerance	CNAG_05063	*SSK2*	Eukaryot Cell. 2007 Dec;6(12):2278-89.
Thermotolerance	CNAG_00769	*PBS2*	Mol Biol Cell. 2005 May;16(5):2285-300.
Thermotolerance	CNAG_00293	*RAS1*	Eukaryot Cell. 2010 Mar;9(3):360-78.
Thermotolerance	CNAG_05968	*CDC420*	Mol Microbiol. 2010 Feb;75(3):763-80.
Thermotolerance	CNAG_03670	*IRE1*	PLoS Pathog. 2011 Aug;7(8):e1002177
Thermotolerance	CNAG_00815	*SIT1*	Microbiology. 2007 Jan;153(Pt 1):29-41.
Thermotolerance	CNAG_04514	*MPK1*	Mol Microbiol. 2003 Jun;48(5):1377-87
Thermotolerance	CNAG_02867	*PLC1*	Mol Microbiol. 2008 Aug;69(4):809-26.

Supplementary Table S3. Predicted top 100 candidate for three virulence factors by guilt-by association (√: tested strain)

Capsule formation

Rank	CNAG_ID	name	Sc ortholog	Score	This study (phenotype)	Madhani's study (phenotype)
1	CNAG_04755	*BCK1*	*BCK1*	27.86		
2	CNAG_04514	*MPK1*	*SLT2*	26.66	√ (WT-level)	√ (WT-level)
3	CNAG_00293	*RAS1*	*RAS2*	24.77		
4	CNAG_01664		*CDC28*	24.09		
5	CNAG_02357	*MKK2*	*MKK2*	20.88		
6	CNAG_05970		*STE20*	20		
7	CNAG_00745		*NPR1*	19.96	√ (WT-level)	√ (WT-level)
8	CNAG_04148		*KIN3*	19.89		
9	CNAG_06552	*SNF1*	*SNF1*	19.85	√ (WT-level)	√ (WT-level)
10	CNAG_00683		*KNS1*	19.81		
11	CNAG_04678	*YPK1*	*YPK1*	19.7		
12	CNAG_02194		*DBF20*	19.66		
13	CNAG_06730	*GSK3*	*RIM11*	19.52		√ (Enhanced)
14	CNAG_01730	*STE7*	*STE7*	19.46	√ (WT-level)	√ (WT-level)
15	CNAG_02531	*CPK2*	*FUS3*	19.44	√ (WT-level)	√ (WT-level)
16	CNAG_07675		*IRA2*	19.36		
17	CNAG_02511	*CPK1*		19	√ (WT-level)	√ (WT-level)
18	CNAG_06568		*SKS1*	18.92	√ (WT-level)	√ (WT-level)
19	CNAG_00130	*HRK1*	*RCK2*	18.87	√ (WT-level)	√ (WT-level)
20	CNAG_03567	*CBK1*	*CBK1*	18.43		
21	CNAG_04040		*FPK1*	18.38	√ (WT-level)	√ (WT-level)
22	CNAG_08022		*PHO85*	18.23		
23	CNAG_06980	*STE11*	*STE11*	17.79		
24	CNAG_04162	*PKA2*		17.55	√ (WT-level)	√ (WT-level)
25	CNAG_01285		*IPL1*	17.36		

26	CNAG_00405	*KIC1*	*KIC1*	17.33		
27	CNAG_07427	*CCK2*	*HRR25*	17.29		
28	CNAG_06086		*SSN3*	17.22	√ (Enhanced)	√ (WT-level)
29	CNAG_03167		*CHK1*	17.22		
30	CNAG_07942		*CDC25*	17.18		
31	CNAG_03811			17.14	√ (Enhanced)	√ (Enhanced)
32	CNAG_02915	*PDK1*	*PKH2*	17.13	√ (WT-level)	√ (WT-level)
33	CNAG_01938	*KIN1*	*KIN1*	17.1	√ (Enhanced)	√ (WT-level)
34	CNAG_05439		*CMK2*	17	√ (WT-level)	√ (WT-level)
35	CNAG_06445		*KIN28*	16.94		
36	CNAG_05216		*RAD53*	16.55	√ (WT-level)	√ (WT-level)
37	CNAG_03290		*SPS1*	16.51	√ (WT-level)	√ (WT-level)
38	CNAG_06490		*YPL150W*	16.36		
39	CNAG_05274			16.19		
40	CNAG_05005		*ATG1*	16.08	√ (WT-level)	√ (WT-level)
41	CNAG_07408	*STE20*	*CLA4*	16.08		
42	CNAG_03409	*SKN7*	*SKN7*	15.89	√ (WT-level)	√ (WT-level)
43	CNAG_05694		*CKA2*	15.52		
44	CNAG_01062		*PSK2*	15.47	√ (WT-level)	√ (WT-level)
45	CNAG_01907		*CDC5*	15.47		
46	CNAG_06809	*IKS1*	*IKS1*	15.31		
47	CNAG_06174		*GCN2*	15.31	√ (WT-level)	√ (WT-level)
48	CNAG_05348	*CDC42*	*CDC42*	15.14		
49	CNAG_06671			15.05	√ (WT-level)	√ (WT-level)
50	CNAG_04118		*CTK1*	14.94		
51	CNAG_06697		*MPS1*	14.93	√ (WT-level)	√ (WT-level)
52	CNAG_00415	*CDC2801*		14.68	√ (WT-level)	√ (WT-level)
53	CNAG_03171	*SWE1*		14.65		
54	CNAG_06845		*CDC15*	14.62	√ (WT-level)	√ (WT-level)
55	CNAG_05484			14.56	√ (WT-level)	√ (WT-level)
56	CNAG_05549		*SGV1*	14.55		

57	CNAG_04433			14.29	√ (WT-level)	√ (WT-level)
58	CNAG_00483	*ACT1*	*ACT1*	14.09		
59	CNAG_06310			14.03		
60	CNAG_00375	*GCN5*	*GCN5*	13.8		
61	CNAG_05499		*SHO1*	13.75		
62	CNAG_02675		*HSL1*	13.6	√ (WT-level)	√ (WT-level)
63	CNAG_07667		*SAT4*	13.27		
64	CNAG_04282	*MPK2*		13.22	√ (WT-level)	√ (WT-level)
65	CNAG_05104			12.98		
66	CNAG_04272			12.96	√ (WT-level)	√ (WT-level)
67	CNAG_05222	*NRG1*	*NRG1*	12.91		√ (Reduced)
68	CNAG_06151	*YPD1*	*YPD1*	12.63		
69	CNAG_02962			12.12		
70	CNAG_00556	*CCK1*	*YCK2*	11.97	√ (WT-level)	√ (WT-level)
71	CNAG_02153	*TUP1*	*TUP1*	11.83		
72	CNAG_04197		*YAK1*	11.5		
73	CNAG_02373		*VPS33*	11.46		
74	CNAG_02820			11.38	√ (WT-level)	√ (WT-level)
75	CNAG_03796		*YPL236C*	11.23	√ (WT-level)	√ (WT-level)
76	CNAG_05558	*KIN4*	*KIN4*	11.08	√ (WT-level)	√ (WT-level)
77	CNAG_04837	*MLN1*	*RTG3*	11.01	√ (WT-level)	√ (WT-level)
78	CNAG_06150		*HSC82*	10.65		
79	CNAG_01682		*TOM70*	10.48		
80	CNAG_01208	*CCH1*	*CCH1*	9.88		
81	CNAG_02563		*POL3*	9.81		
82	CNAG_05581	*CHS3*	*CHS3*	9.58		
83	CNAG_03258			9.52	√ (WT-level)	√ (WT-level)
84	CNAG_03369		*SWE1*	9.43	√ (WT-level)	√ (WT-level)
85	CNAG_05734		*SLA1*	9.29		
86	CNAG_02389			9.18	√ (WT-level)	√ (WT-level)
87	CNAG_04959		*TLG2*	9.1		

88	CNAG_00121		*GPD1*	9.07		
89	CNAG_05155		*PTP3*	8.87		
90	CNAG_03024		*RIM15*	8.76		
91	CNAG_07819			8.75		
92	CNAG_01371	*CRG2*	*RAX1*	8.72		
93	CNAG_03143	*HSP12*	*HSP12*	8.59		
94	CNAG_01820	*PYK1*	*CDC19*	8.55		
95	CNAG_06812	*SPO14α*	*SPO14*	8.51		
96	CNAG_04758	*AMT2*	*MEP2*	8.5		
97	CNAG_00179	*GPA2*		8.44	√ (WT-level)	√ (WT-level)
98	CNAG_04049			8.35		
99	CNAG_05563	*HOS2*	*HOS2*	8.28	√ (Enhanced)	√ (Enhanced)
100	CNAG_04369			8.2		

Melanin

Rank	CNAG_ID	name	Sc ortholog	Score	This study (phenotype)	Madhani's study (phenotype)
1	CNAG_04755	*BCK1*	*BCK1*	32.74		
2	CNAG_06301	*SCH9*	*SCH9*	31.32	√ (WT-level)	√ (WT-level)
3	CNAG_04514	*MPK1*	*SLT2*	30.71	√ (WT-level)	√ (WT-level)
4	CNAG_01664		*CDC28*	27.73		
5	CNAG_04148		*KIN3*	23.46		
6	CNAG_08022		*PHO85*	23.06		
7	CNAG_02531	*CPK2*	*FUS3*	22.93	√ (WT-level)	√ (WT-level)
8	CNAG_04678	*YPK1*	*YPK1*	22.7		
9	CNAG_06730	*GSK3*	*RIM11*	22.48		
10	CNAG_06980	*STE11*	*STE11*	22		
11	CNAG_06568		*SKS1*	21.96	√ (WT-level)	√ (WT-level)
12	CNAG_01285		*IPL1*	21.94		
13	CNAG_02194		*DBF20*	21.83		
14	CNAG_03167		*CHK1*	21.7		

15	CNAG_02915	*PDK1*	*PKH2*	21.68	√ (Reduced)	√ (WT-level)
16	CNAG_05970		*STE20*	21.6		
17	CNAG_00293	*RAS1*	*RAS2*	21.58		
18	CNAG_05216		*RAD53*	21.57	√ (WT-level)	√ (WT-level)
19	CNAG_02511	*CPK1*		21.5	√ (WT-level)	√ (WT-level)
20	CNAG_06086		*SSN3*	21.49	√ (Reduced)	√ (WT-level)
21	CNAG_03567	*CBK1*	*CBK1*	20.83		
22	CNAG_01938	*KIN1*	*KIN1*	20.64	√ (Enhanced)	√ (WT-level)
23	CNAG_01907		*CDC5*	20.53		
24	CNAG_01730	*STE7*	*STE7*	20.52	√ (WT-level)	√ (WT-level)
25	CNAG_00683		*KNS1*	20.46		
26	CNAG_00745		*NPR1*	20.43	√ (WT-level)	√ (WT-level)
27	CNAG_00405	*KIC1*	*KIC1*	20.15		
28	CNAG_03290		*SPS1*	20.12	√ (Enhanced)	√ (WT-level)
29	CNAG_06445		*KIN28*	20.04		
30	CNAG_04040		*FPK1*	19.99	√ (WT-level)	√ (WT-level)
31	CNAG_05439		*CMK2*	19.56	√ (WT-level)	√ (WT-level)
32	CNAG_06490		*YPL150W*	19.44		
33	CNAG_06845		*CDC15*	19.36	√ (WT-level)	√ (WT-level)
34	CNAG_05005		*ATG1*	19.12	√ (WT-level)	√ (WT-level)
35	CNAG_03811			19.08	√ (WT-level)	√ (WT-level)
36	CNAG_01062		*PSK2*	18.83	√ (WT-level)	√ (WT-level)
37	CNAG_05694		*CKA2*	18.57		
38	CNAG_06809	*IKS1*	*IKS1*	18.36		
39	CNAG_07408	*STE20*	*CLA4*	18.22		
40	CNAG_05484			18.19	√ (WT-level)	√ (WT-level)
41	CNAG_05274			18.16		
42	CNAG_04162	*PKA2*		18.11	√ (WT-level)	√ (WT-level)
43	CNAG_06671			18.07	√ (WT-level)	√ (WT-level)
44	CNAG_03171	*SWE1*		17.97		

45	CNAG_04118		*CTK1*	17.83			
46	CNAG_06697		*MPS1*	17.75	√ (WT-level)	√ (WT-level)	
47	CNAG_00415	*CDC2801*		17.46		√ (Reduced)	
48	CNAG_05549		*SGV1*	17.28			
49	CNAG_06310			17.24			
50	CNAG_02675		*HSL1*	17.22	√ (WT-level)	√ (WT-level)	
51	CNAG_04282	*MPK2*		16.82	√ (WT-level)	√ (WT-level)	
52	CNAG_03670	*IRE1*	*IRE1*	16.61			
53	CNAG_07427	*CCK2*	*HRR25*	16.57			
54	CNAG_04272			15.95	√ (WT-level)	√ (WT-level)	
55	CNAG_06174		*GCN2*	15.58	√ (WT-level)	√ (WT-level)	
56	CNAG_00556	*CCK1*	*YCK2*	15.56		√ (Reduced)	
57	CNAG_07667		*SAT4*	15.17			
58	CNAG_05348	*CDC42*	*CDC42*	15.15			
59	CNAG_07675		*IRA2*	15.1			
60	CNAG_02962			15.03			
61	CNAG_00570	*PKR1*	*BCY1*	14.58			
62	CNAG_04433			14.58	√ (WT-level)	√ (WT-level)	
63	CNAG_05104			14.45			
64	CNAG_01682		*TOM70*	14.34			
65	CNAG_05499		*SHO1*	13.93			
66	CNAG_03315	*RHO1*	*RHO1*	13.75			
67	CNAG_03796		*YPL236C*	13.59	√ (WT-level)	√ (WT-level)	
68	CNAG_05155		*PTP3*	13.54			
69	CNAG_06151	*YPD1*	*YPD1*	12.95			
70	CNAG_02153	*TUP1*	*TUP1*	12.85			
71	CNAG_02820			12.79	√ (WT-level)	√ (WT-level)	
72	CNAG_05222	*NRG1*	*NRG1*	12.77			
73	CNAG_04197		*YAK1*	12.64			
74	CNAG_02373		*VPS33*	12.18			
75	CNAG_03369		*SWE1*	11.85	√ (WT-level)	√ (WT-level)	

76	CNAG_04049			11.69		
77	CNAG_02389			11.5	√ (WT-level)	√ (WT-level)
78	CNAG_05558	*KIN4*	*KIN4*	11.12	√ (WT-level)	√ (WT-level)
79	CNAG_07942		*CDC25*	10.97		
80	CNAG_02028		*SKY1*	10.6		
81	CNAG_04837	*MLN1*	*RTG3*	10.46		√ (Reduced)
82	CNAG_03258			10.38	√ (WT-level)	√ (WT-level)
83	CNAG_03024		*RIM15*	10.36		
84	CNAG_02185		*YNL022C*	10.23		
85	CNAG_03791		*SAK1*	10.21		
86	CNAG_01208	*CCH1*	*CCH1*	10.12		
87	CNAG_01347			10.02		
88	CNAG_05704		*SNF8*	10.02		
89	CNAG_01612			9.95		
90	CNAG_03143	*HSP12*	*HSP12*	9.79		
91	CNAG_01988	*TCO3*	*SLN1*	9.79	√ (WT-level)	√ (WT-level)
92	CNAG_00782			9.69	√ (WT-level)	√ (WT-level)
93	CNAG_00483	*ACT1*	*ACT1*	9.61		
94	CNAG_06642	*TOR1*	*TOR2*	9.53		
95	CNAG_01902		*CYC8*	9.13		
96	CNAG_05734		*SLA1*	8.81		
97	CNAG_03706		*GLC7*	8.81		
98	CNAG_03998	*RLM1*	*RLM1*	8.67	√ (WT-level)	√ (WT-level)
99	CNAG_00537		*CAT2*	8.59		
100	CNAG_05932		*CPR6*	8.41		

Thermotolerance

Rank	CNAG_ID	name	Sc ortholog	Score	This study (phenotype)	Madhani's study (phenotype)
1	CNAG_05970		*STE20*	29.79		
2	CNAG_01664		*CDC28*	27.97		

3	CNAG_02531	*CPK2*	*FUS3*	25.99		√ (Reduced)
4	CNAG_00396	*PKA1*	*TPK1*	25.42	√ (WT-level)	√ (WT-level)
5	CNAG_07408	*STE20*	*CLA4*	25.3		
6	CNAG_08022		*PHO85*	25.25		
7	CNAG_02511	*CPK1*		25.1	√ (WT-level)	√ (WT-level)
8	CNAG_04678	*YPK1*	*YPK1*	24.55		
9	CNAG_01938	*KIN1*	*KIN1*	24.31	√ (Reduced)	√ (WT-level)
10	CNAG_06086		*SSN3*	24.27	√ (WT-level)	√ (WT-level)
11	CNAG_01730	*STE7*	*STE7*	23.75	√ (WT-level)	√ (WT-level)
12	CNAG_00130	*HRK1*	*RCK2*	23.57	√ (WT-level)	√ (WT-level)
13	CNAG_06730	*GSK3*	*RIM11*	23.18		
14	CNAG_03409	*SKN7*	*SKN7*	23.04		√ (Reduced)
15	CNAG_04148		*KIN3*	22.89		
16	CNAG_03171	*SWE1*		22.81		
17	CNAG_03567	*CBK1*	*CBK1*	22.67		
18	CNAG_01285		*IPL1*	22.58		
19	CNAG_06980	*STE11*	*STE11*	22.31		
20	CNAG_02915	*PDK1*	*PKH2*	22.1	√ (Reduced)	√ (Reduced)
21	CNAG_00405	*KIC1*	*KIC1*	22.06		
22	CNAG_03167		*CHK1*	22.02		
23	CNAG_03290		*SPS1*	21.97	√ (Reduced)	√ (Reduced)
24	CNAG_02194		*DBF20*	21.92		
25	CNAG_05499		*SHO1*	21.88		
26	CNAG_06552	*SNF1*	*SNF1*	21.72	√ (Reduced)	√ (WT-level)
27	CNAG_05216		*RAD53*	21.69	√ (WT-level)	√ (WT-level)
28	CNAG_06445		*KIN28*	21.68		
29	CNAG_04040		*FPK1*	21.47	√ (WT-level)	√ (WT-level)
30	CNAG_05439		*CMK2*	21.37	√ (WT-level)	√ (WT-level)
31	CNAG_01907		*CDC5*	21.2		
32	CNAG_03811			21.15		√ (Reduced)
33	CNAG_06490		*YPL150W*	20.8		

34	CNAG_06568		*SKS1*	20.77	√ (WT-level)	√ (WT-level)
35	CNAG_00683		*KNS1*	20.58		
36	CNAG_06671			20.35	√ (WT-level)	√ (WT-level)
37	CNAG_07427	*CCK2*	*HRR25*	20.26		
38	CNAG_05005		*ATG1*	20.23	√ (WT-level)	√ (WT-level)
39	CNAG_01062		*PSK2*	19.78	√ (WT-level)	√ (WT-level)
40	CNAG_03315	*RHO1*	*RHO1*	19.71		
41	CNAG_04118		*CTK1*	19.54		√ (Reduced)
42	CNAG_06697		*MPS1*	19.41	√ (Reduced)	√ (WT-level)
43	CNAG_05549		*SGV1*	19.4		
44	CNAG_00415	*CDC2801*		19.38	√ (Reduced)	√ (WT-level)
45	CNAG_06845		*CDC15*	19.35	√ (Reduced)	√ (Reduced)
46	CNAG_04162	*PKA2*		19.22	√ (WT-level)	√ (WT-level)
47	CNAG_04505	*GPA1*	*GPA2*	19.09		
48	CNAG_02675		*HSL1*	18.79	√ (Reduced)	√ (Reduced)
49	CNAG_04282	*MPK2*		18.46		√ (Reduced)
50	CNAG_05484			18.24	√ (WT-level)	√ (WT-level)
51	CNAG_05274			18.1		
52	CNAG_05694		*CKA2*	18.04		
53	CNAG_06809	*IKS1*	*IKS1*	17.97		
54	CNAG_06310			17.92		
55	CNAG_00745		*NPR1*	16.74	√ (WT-level)	√ (WT-level)
56	CNAG_04272			16.73	√ (WT-level)	√ (WT-level)
57	CNAG_07667		*SAT4*	16.45		
58	CNAG_06174		*GCN2*	16.45	√ (WT-level)	√ (WT-level)
59	CNAG_00556	*CCK1*	*YCK2*	16.04	√ (Reduced)	√ (WT-level)
60	CNAG_03998	*RLM1*	*RLM1*	15.69	√ (WT-level)	√ (WT-level)
61	CNAG_05104			15.54		
62	CNAG_02962			15.4		
63	CNAG_06151	*YPD1*	*YPD1*	15.36		

64	CNAG_03796		*YPL236C*	15.11	√ (WT-level)	√ (WT-level)
65	CNAG_04433			14.26	√ (WT-level)	√ (WT-level)
66	CNAG_02820			14.17	√ (WT-level)	√ (WT-level)
67	CNAG_04119	*ROM2*	*ROM2*	13.69		
68	CNAG_05838	*RGD1*	*RGD1*	13.62	√ (WT-level)	√ (WT-level)
69	CNAG_01682		*TOM70*	12.96		
70	CNAG_05558	*KIN4*	*KIN4*	12.77	√ (Reduced)	√ (Reduced)
71	CNAG_04197		*YAK1*	12.77		
72	CNAG_04049			12.7		
73	CNAG_05581	*CHS3*	*CHS3*	12.61		
74	CNAG_03369		*SWE1*	12.26	√ (WT-level)	√ (WT-level)
75	CNAG_03258			12.17	√ (WT-level)	√ (WT-level)
76	CNAG_04090	*ATF1*		12.11		
77	CNAG_02373		*VPS33*	11.99		
78	CNAG_06501		*GAS1*	11.13		
79	CNAG_00888	*CNB1*	*CNB1*	11.01		
80	CNAG_05734		*SLA1*	10.98		
81	CNAG_02389			10.98	√ (WT-level)	√ (WT-level)
82	CNAG_05081	*PDE1*	*PDE1*	10.93		
83	CNAG_06597	*SPT8*	*SPT8*	10.8		
84	CNAG_01612			10.8		
85	CNAG_06642	*TOR1*	*TOR2*	10.71		
86	CNAG_04837	*MLN1*	*RTG3*	10.64	√ (WT-level)	√ (WT-level)
87	CNAG_06508	*FKS1*	*GSC2*	10.61		
88	CNAG_03024		*RIM15*	10.55		
89	CNAG_02028		*SKY1*	10.53		
90	CNAG_05218	*ACA1*	*SRV2*	10.51		
91	CNAG_05155		*PTP3*	10.21		
92	CNAG_01208	*CCH1*	*CCH1*	10.19		
93	CNAG_04815	*BNI1*	*BNI1*	10.12		
94	CNAG_03345	*SSD1*	*SSD1*	9.73		

95	CNAG_01347			9.38		
96	CNAG_05222	*NRG1*	*NRG1*	9.28		
97	CNAG_00782			9.14	√ (WT-level)	√ (WT-level)
98	CNAG_03967		*CAP1*	8.91		√ (Reduced)
99	CNAG_02185		*YNL022C*	8.86		
100	CNAG_06898		*CHS7*	8.65		

Supplementary Table S4. 230 upregulated genes upon fluconazol treatment (> 2 fold change)

H99 locus ID	Gene name	Fold change
CNAG_03644	*CAS3*	11.60743947
CNAG_01737	*ERG25*	4.100518739
CNAG_03007		9.893845024
CNAG_03204		4.961017102
CNAG_02896	*ERG130*	3.435417488
CNAG_00854	*ERG2*	4.025915577
CNAG_04280		5.861502749
CNAG_02226		5.406848223
CNAG_02701		5.866838761
CNAG_01841	*GLN3*	4.971495898
CNAG_07486		4.266962877
CNAG_07492		6.45571552
CNAG_01856		3.445989571
CNAG_01750		2.835550804
CNAG_03122		2.730722453
CNAG_05159		3.481718653
CNAG_07596		3.586669867
CNAG_06020		2.884107728
CNAG_00699		4.907805416
CNAG_02523		2.763268524
CNAG_03238		3.15748165
CNAG_02776		3.207504667
CNAG_03848		4.060469166
CNAG_06336		2.604440066
CNAG_07450		2.45835813
CNAG_04934		2.509589739
CNAG_03176		2.500790953
CNAG_03461		5.522200944

CNAG_03974		3.112888768
CNAG_05338		2.440693079
CNAG_05545		2.261789837
CNAG_07448	*DUR3*	4.66774394
CNAG_05258		4.820232874
CNAG_05113		2.366303137
CNAG_04632		5.792334022
CNAG_02389		2.805017954
CNAG_00519	*ERG3*	2.30839147
CNAG_01742	*AQY1*	3.880759245
CNAG_02797	*CPL1*	3.004409816
CNAG_06443	*KAR2*	2.329963569
CNAG_06482		2.25129493
CNAG_04758	*AMT2*	3.210331716
CNAG_00679		3.714772521
CNAG_06347	*BLP2*	6.837556225
CNAG_04675		3.435086428
CNAG_00895	*ZIP1*	2.160317283
CNAG_03857		2.224809082
CNAG_07731		3.416125193
CNAG_01207		3.1057044
CNAG_06829	*ERG1*	2.704018074
CNAG_01090		2.238673309
CNAG_01796		3.94460594
CNAG_03999		3.170201929
CNAG_04210		2.245062609
CNAG_06266		2.233119834
CNAG_06509		4.243572986
CNAG_06424		2.524879793
CNAG_02990		2.350044469
CNAG_04747		2.158331318

CNAG_04782		2.153770613
CNAG_03913		2.534772224
CNAG_03294		2.180905748
CNAG_02156		2.346358666
CNAG_02446		2.419291822
CNAG_05453		2.593700242
CNAG_03078		2.006918961
CNAG_03316	*RDI1*	2.014463256
CNAG_05458		3.250183492
CNAG_00544		2.032973435
CNAG_02552		2.805682916
CNAG_04313		2.005395196
CNAG_05521		2.09220702
CNAG_00040	*ERG11*	3.060460867
CNAG_05678		2.570092635
CNAG_02531	*CPK2*	2.478999734
CNAG_00696		2.956934211
CNAG_05592	*ECA1*	2.590543496
CNAG_00407		2.33564059
CNAG_06785		2.156432684
CNAG_05023	*CAS91*	2.063796764
CNAG_02176		2.497828778
CNAG_04017		2.69421758
CNAG_06644	*ERG5*	3.019376085
CNAG_07633		2.304366491
CNAG_02225	*EXG104*	3.47656117
CNAG_06652		2.366321132
CNAG_03664	*NIC1*	2.128492371
CNAG_02440		2.295752063
CNAG_00038		2.018871609
CNAG_04681		2.395168859

CNAG_01861		2.072645093
CNAG_00419		2.86195785
CNAG_07902		2.419220693
CNAG_02830	*ERG4*	2.420948502
CNAG_01129	*ERG7*	2.549941476
CNAG_03385	*PCL103*	2.283950008
CNAG_00751		3.111496815
CNAG_01070		2.859000112
CNAG_06863		2.0519339
CNAG_03400		2.908045958
CNAG_05841		2.070408262
CNAG_00691		2.205021879
CNAG_00605		2.13554549
CNAG_00302		2.198066632
CNAG_06331		2.300082232
CNAG_02918	*ERG10*	2.449583486
CNAG_04635		2.379529269
CNAG_04874		2.812216398
CNAG_02964		2.061018137
CNAG_03163		2.402112615
CNAG_04652		2.432521954
CNAG_00818		2.000727327
CNAG_03326	*CHS2*	2.41060654
CNAG_00247	*LYS9*	2.021856778
CNAG_05607		2.662405297
CNAG_04553		3.27177622
CNAG_04804	*SRE1*	5.564225285
CNAG_05736		2.285635428
CNAG_00331		2.447695837
CNAG_02718		2.176061773
CNAG_05932		2.753627491

CNAG_00132		2.727910719
CNAG_02848		2.183183028
CNAG_00838		2.428786635
CNAG_01718		2.057848977
CNAG_06727		2.175837382
CNAG_05289		2.245503035
CNAG_02217	*CHS7*	2.639549802
CNAG_04413		3.156348677
CNAG_02297		2.294933532
CNAG_04862		2.071920132
CNAG_03651		2.003144033
CNAG_03816		2.452008141
CNAG_07943		2.679043773
CNAG_04640	*ACL1*	2.053883538
CNAG_02308		2.101464305
CNAG_06296		3.93552104
CNAG_02735		4.903857337
CNAG_07337		2.028760911
CNAG_01131		2.027670489
CNAG_02309		2.11137462
CNAG_00117	*ERG24*	2.436478079
CNAG_05005		2.326199159
CNAG_07476		2.467123277
CNAG_01736		2.274198065
CNAG_05778		2.000546899
CNAG_03698		2.034237514
CNAG_03709		2.204269454
CNAG_07463		2.406976199
CNAG_02062		2.314773587
CNAG_02052		2.589101725
CNAG_05024		2.058801357

CNAG_01706		2.013315237
CNAG_00652		2.977733917
CNAG_00164		2.268927265
CNAG_01695		2.142598003
CNAG_03754		2.456715849
CNAG_00157		2.003158066
CNAG_01957		2.020821188
CNAG_05252		2.042608164
CNAG_00454		2.643050182
CNAG_02934		3.052457927
CNAG_04084		2.307490961
CNAG_03237		2.000906047
CNAG_03856		2.80503012
CNAG_02500		2.12603782
CNAG_03346		2.019855655
CNAG_00598		2.173745452
CNAG_00023		2.630398477
CNAG_03560		2.099762767
CNAG_03021		2.641718848
CNAG_05870		2.101227513
CNAG_05558	*KIN4*	2.197384541
CNAG_04832		2.350465716
CNAG_00801		2.562856914
CNAG_02222		2.380768794
CNAG_02192		2.004619275
CNAG_05256	*CAT2*	2.117893164
CNAG_05541		2.027979224
CNAG_05443		2.139557938
CNAG_05192		2.554092586
CNAG_04873		2.047412618
CNAG_04801		2.48505801

CNAG_04589		2.200649668
CNAG_07493		2.23564484
CNAG_01244		2.220645594
CNAG_02497		2.277640498
CNAG_06808	*CPRa*	2.331898848
CNAG_03598		2.105246993
CNAG_06787		2.141358214
CNAG_00905		2.436259285
CNAG_03828		2.864423668
CNAG_03464	*LAC2*	2.119531919
CNAG_04975		2.123979621
CNAG_01314		2.152250629
CNAG_05379		2.328096422
CNAG_05638		2.544322173
CNAG_06245		2.20108488
CNAG_01992		2.005703165
CNAG_01034		2.522909142
CNAG_00484		2.216837227
CNAG_03639		2.026990697
CNAG_04858		2.079882603
CNAG_03486		2.000019906
CNAG_05188		2.613416987
CNAG_05020		2.621197783
CNAG_05992		2.437276546
CNAG_07843		2.511746725
CNAG_06540		2.723356183
CNAG_00261		2.279401635
CNAG_06931		2.409513121
CNAG_05294		2.216230896
CNAG_05001		2.083402951
CNAG_04333		2.014859926

CNAG_01194		2.485825691
CNAG_03944		2.150102862
CNAG_04642		2.125378145
CNAG_04562		2.054816681
CNAG_06365		2.175916961
CNAG_07380		5.265638134
CNAG_00376		2.046841951
CNAG_00887		3.02481325
CNAG_05093		2.271206696
CNAG_07909		2.012455842
CNAG_06133		2.144202545
CNAG_01827		2.23421932
CNAG_01689		2.432645037
CNAG_04711		2.782921282
CNAG_03337		2.154466763
CNAG_01672		2.19161274

Supplementary Table S5. Predicted top 94 candidates (P-value < 0.05) as context-associated hub genes (16 genes involved in ergosterol pathway; 2 genes identified at PMID-22339665; 1 gene identified at PMID-19700638); DEG: Differentially Expressed Gene; √: tested strains

Rank	CNAG_ID	name	Sc ortholog	P-value	Submitted DEG	Test strain
1	CNAG_04605	*ERG26*	*ERG26*	2.44E-07		
2	CNAG_03311	*ERG13*	*ERG13*	1.55E-06		
3	CNAG_00519	*ERG3*	*ERG3*	5.90E-06	DEG	
4	CNAG_02363		*FLR1*	8.55E-06		√
5	CNAG_01003	*NCP1*	*NCP1*	1.17E-05		
6	CNAG_03176		*ERO1*	5.74E-05	DEG	
7	CNAG_01129	*ERG7*	*ERG7*	7.72E-05	DEG	
8	CNAG_06829	*ERG1*	*ERG1*	9.59E-05	DEG	
9	CNAG_04238		*SPS19*	0.000125129		
10	CNAG_00854	*ERG2*	*ERG2*	0.000420586	DEG	
11	CNAG_02084	*ERG20*	*ERG20*	0.000458534		
12	CNAG_00115		*SOR1*	0.000480706		
13	CNAG_06240		*PDI1*	0.000590007		
14	CNAG_07448	*DUR3*	*DUR3*	0.000642028	DEG	
15	CNAG_03486		*CPR5*	0.000707427	DEG	
16	CNAG_02361		*FEN2*	0.000805704		
17	CNAG_03899		*LHS1*	0.000805704		
18	CNAG_00305		*AHA1*	0.000819508		
19	CNAG_02830	*ERG4*	*ERG4*	0.001146385	DEG	
20	CNAG_00060			0.001152911		
21	CNAG_00869	*PDR5*		0.00117732		√
22	CNAG_03618		*ZTA1*	0.001310014		
23	CNAG_05137		*ERD2*	0.001460349		
24	CNAG_02918	*ERG10*	*ERG10*	0.001487869	DEG	
25	CNAG_01737	*ERG25*	*ERG25*	0.001920833	DEG	
26	CNAG_03009	*ERG28*	*ERG28*	0.002726871		
27	CNAG_06901	*ALG7*	*ALG7*	0.002776791		
28	CNAG_04659		*PDC1*	0.003010344		
29	CNAG_02460	*HEM13*	*HEM13*	0.003379026		
30	CNAG_00539		*TPO2*	0.003853456		
31	CNAG_06835	*KRE61*	*KRE6*	0.004446774		
32	CNAG_06534	*HMG1*	*HMG2*	0.004642301		
33	CNAG_03939		*HEM1*	0.004642301		
34	CNAG_06644	*ERG5*	*ERG5*	0.004795121	DEG	
35	CNAG_05252		*SCJ1*	0.004845469	DEG	

36	CNAG_02365		*AMD2*	0.006255902		
37	CNAG_03485		*DSK2*	0.006702894	DEG	√
38	CNAG_03598			0.006702894		√
39	CNAG_01955			0.007343171		
40	CNAG_05125		*MVD1*	0.007936506		
41	CNAG_01696			0.008057294		
42	CNAG_01347			0.009380254		
43	CNAG_00040	*ERG11*	*ERG11*	0.009461903	DEG	
44	CNAG_06241	*CFO1*	*FET5*	0.009867204		
45	CNAG_02309		*FPR2*	0.009867204	DEG	
46	CNAG_05136		*SPF1*	0.010273188		
47	CNAG_06898		*CHS7*	0.012046246		
48	CNAG_04678	*YPK1*	*YPK1*	0.012209923		
49	CNAG_00515			0.012622739		
50	CNAG_05090			0.012622739		√
51	CNAG_07524		*AMD1*	0.012622739		
52	CNAG_03079	*PER1*	*PER1*	0.013571067		
53	CNAG_02777	*PHO84*	*PHO84*	0.013800327		
54	CNAG_01076		*UGA1*	0.014026546		
55	CNAG_07561		*GND1*	0.014422346		
56	CNAG_02225	*EXG104*	*EXG1*	0.01596303	DEG	
57	CNAG_08012		*VMA3*	0.017081865		
58	CNAG_04514	*MPK1*	*SLT2*	0.018869409		√
59	CNAG_06431			0.020392163		√
60	CNAG_01615			0.022522129		
61	CNAG_01201		*MET10*	0.024231447		√
62	CNAG_03168		*NCB2*	0.024231447		
63	CNAG_02500		*CNE1*	0.025614652	DEG	
64	CNAG_00815	*SIT1*	*SIT1*	0.027295229		
65	CNAG_00816		*ATG8*	0.027295229		
66	CNAG_05932		*CPR6*	0.027683881	DEG	
67	CNAG_02455		*HNM1*	0.028718526		
68	CNAG_02604		*HOP1*	0.029648344		
69	CNAG_05538	*JJJ1*	*JJJ1*	0.029787843		√
70	CNAG_02489			0.032665171		
71	CNAG_00800		*PNC1*	0.032695017		
72	CNAG_01464	*FHB1*	*YHB1*	0.033951984		
73	CNAG_00711			0.034105583		√
74	CNAG_03834		*SUR2*	0.034105583		
75	CNAG_06121			0.034105583		

76	CNAG_06361		*RPN6*	0.035357005		
77	CNAG_00383	*APT3*	*DRS2*	0.035726905		
78	CNAG_07779			0.036481894		
79	CNAG_00062		*STE7*	0.037328278		
80	CNAG_01730	*STE7*	*RPN3*	0.037328278		√
81	CNAG_03358		*PGK1*	0.037328278		
82	CNAG_00064		*EMP24*	0.038745979		
83	CNAG_06917	*TSA3*	*PRX1*	0.040461901		
84	CNAG_06927			0.041533921		
85	CNAG_01861		*NAS6*	0.042420381	DEG	
86	CNAG_00490		*POT1*	0.042492503		
87	CNAG_04388	*SOD2*	*SOD2*	0.042685966		
88	CNAG_00747		*LSC2*	0.043151587		
89	CNAG_04677			0.044210882		
90	CNAG_05657			0.044210882		
91	CNAG_02484			0.045467679		
92	CNAG_04651		*ERV29*	0.045467679		
93	CNAG_05792		*PRE9*	0.048055995		
94	CNAG_02761		*PHO88*	0.049898183		

Supplementary Table S6. Inferred GO-BP terms of CNAG_00711 from its network neighbors

Rank	Score	Evidences	Sc GO biological process terms	GO biological process terms_supporters(LLS)
1	1.5	CN-CX:1.00	NADH oxidation	*GPD1* (1.50)
2	1.5	CN-CX:1.00	intracellular accumulation of glycerol	*GPD1* (1.50)
3	1.42	CN-CX:1.00	nucleotide metabolic process	*HNT1* (1.42)
4	1.27	CN-CX:1.00	D-amino acid metabolic process	*HPA3* (1.27)
5	1.27	CN-CX:1.00	D-xylose catabolic process	*YJR096W* (1.27)
6	1.27	CN-CX:1.00	arabinose catabolic process	*YJR096W* (1.27)
7	1.27	CN-CX:1.00	cellular response to oxidative stress	*YJR096W* (1.27)
8	1.24	CN-CX:1.00	base-excision repair, AP site formation	*MAG1* (1.24)
9	1.24	CN-CX:1.00	DNA dealkylation involved in DNA repair	*MAG1* (1.24)
10	1.19	CN-CX:1.00	transcription from RNA polymerase I promoter	*RPO26* (1.19)

Supplementary Table S7. Functional descriptions of novel genes for *C. neoformans* virulence

Gene	Functional description
HOS2	Deletion increased capsule production. It encodes a histone deacetylase and is implicated in transcriptional regulation in *S. cerevisiae* [1-3]. Recently, the SAGA histone acetyltransferase complex has been reported to affect the capsule production in *C. neoformans* [4,5], indicating that histone acetylation/deacetylation might be indirectly involved in capsule production of *C. neoformans* by regulating other capsule-related genes.
YPK1	Deletion significantly influences on melanin and capsule production and thermotolerance, and thereby abolishes the virulence of *C. neoformans*. In *S. cerevisiae*, Ypk1 plays roles in sphingolipid synthesis and receptor-mediated endocytosis [6,7]. Similarly, the deletion of *YPK1* leads to the decreased sphingolipid contents, thereby showing reduced cell viability in response to fluconazole in *C. neoformans* [8].
PDK1	It encodes a phosphoinositide-dependent kinase 1. Deletion severely impaired melanin production and growth at high temperature, which may result in virulence attenuation. Pdk1 has been reported to play roles in tolerance to azole drugs and high salt stress and *in vivo* virulence [8,9].
SSN3	Deletion repressed melanin biosynthesis but promoted capsule production. This might be the reason why the *ssn3Δ* mutant was as virulent as the wild-type strain, Ssn3 is a cyclin-dependent protein kinase, which is a component of RNA polymerase II holoenzyme and involved in diverse stress responses including thermosensitivity in *S. cerevisiae* [10,11]. In *C. albicans*, Ssn3 regulates the expression of iron uptake genes through the Sef1 transcription factor and affects its virulence [12,13].
CDC2801	It is predicted to encode Ser/Thr and Tyr kinases, and highly homologous to *S. cerevisiae* Cdc28 (298 aa) encoding cyclin-dependent kinase catalytic subunit. However, CNAG_01664 appears to be a true Cdc28 ortholog (298 aa).
CNAG_03811	It is predicted to encode Ser/Thr and Tyr kinases. Although CNAG_03801 (484 aa) is highly homologous to *S. cerevisiae* Tpk3 (398 aa) encoding catalytic subunits of cAMP-dependent protein kinase A, CNAG_00396 (Pka1, 515 aa) is more homologous to Tpk3.

Supplementary Table S8. GEO expression data sets incorporated into CryptoNet

GEO series	Title	#samples	#node	#edge
GSE6226	Hypoxia response in *C. neoformans* in H99, CM092 and CM098	16	2101	34499
GSE11390	$CoCl_2$ Response in *Cryptococcus neoformans*	8	1274	41517
GSE16692	Remodeling of global transcription patterns of *Cryptococcus neoformans* genes mediated by the stress-activated HOG signaling pathways	100	4312	214974
GSE21176	Molecular and Functional Characterization of the role of RNA silencing components in *Cryptococcus neoformans* [RNAi_Serotype A]	16	1613	30468
GSE21192	Comparative transcriptome analysis of the CO_2 sensing pathway via differential expressions of carbonic anhydrase in *Cryptococcus neoformans*	17	2174	56489
GSE31911	Cryptococcal H99 cells grown in 8 conditions for capsule induction	22	3406	46530
GSE43370	Global transcriptome analysis of eukaryotic genes affected by Gromwell extract	12	849	15522

Supplementary Table S9. Primers used in this study

Primer Name	Sequence (5'-3')	Comment
B79	TGTGGATGCTGGCGGAGGATA	Screening primer on ACT promoter
B1026	GTAAAACGACGGCCAGTGAGC	M13 forward (extended)
B1027	CAGGAAACAGCTATGACCATG	M13 reverse (extended)
B1454	AAGGTGTTCCCCGACGACGAATCG	NSL2
B1455	AACTCCGTCGCGAGCCCCATCAAC	NSR2
B4213	TGAGGTGGAGGCTTGTCTAC	*KIN1* - 5' screening primer
B4209	AGAGACAAAGGTGAGGTCG	*KIN1* - left flanking primer 1
B4210	TCACTGGCCGTCGTTTTACCACGGGATAATGTTGACG	*KIN1* - left flanking primer 2
B4211	CATGGTCATAGCTGTTTCCTGGCAGTATCAAATGCTGGC	*KIN1* - right flanking primer 1
B4212	AGATAATAAGGGTGCGGC	*KIN1* - right flanking primer 2
B4214	GGACTTCTTTGGTTGGGAG	*KIN1* – Southern probe primer 1
B4203	AACGAACGGAAGATTGGC	*SHO1* - 5' screening primer
B4204	ATCTCCCAATCTCCCGAAG	*SHO1* - left flanking primer 1
B4205	TCACTGGCCGTCGTTTTACAAGAAAGACTGGGTGTCGC	*SHO1* - left flanking primer 2
B4206	CATGGTCATAGCTGTTTCCTGACACCCGCTGGTATTACAG	*SHO1* - right flanking primer 1
B4207	AAGTTTTCCTCCACTCGCC	*SHO1* - right flanking primer 2
B4208	GCTGCTTACTACATCTGGACG	*SHO1* – Southern probe primer 1
B4173	TACCCATCATTCCCTGCTC	*YPK1* - 5' screening primer
B4169	CGACTATGGGTTCGTTACTGG	*YPK1* - left flanking primer 1
B4170	TCACTGGCCGTCGTTTTACTGTCTATGCGTTTTCCGAC	*YPK1* - left flanking primer 2
B4171	CATGGTCATAGCTGTTTCCTGTGGTGTAGAATGGCAGAGC	*YPK1* - right flanking primer 1
B4172	GCACCGTGGAGGTAGTAATG	*YPK1* - right flanking primer 2
B4174	ACACCGTATCAGCACAAGC	*YPK1* – Southern probe primer 1

Supplementary Methods

Sequence and functional annotation data for *Cryptococcus neoformans*

CryptoNet was constructed for the all protein coding genes of *C. neoformans* var. *grubii* H99 (serotype A) which lists 6,962 genes validated by deep coverage RNA-sequences [14]. We also added 13 validated mitochondria coding genes for further coverage (*C. neoformans var. grubii* H99 Sequencing Project, Broad Institute of Harvard and MIT, http://www.broadinstitute.org). As a result, a total of 6,975 protein coding genes were used for the construction of a *C. neoformans* functional network. For functional analysis of *C. neoformans* genes, we used Gene Ontology (GO) annotations for *Saccharomyces cerevisiae* orthologs, obtained from Saccharomyces Genome Database (SGD) [15]. Orthologous genes between two species are assigned by BLASTP bidirectional best hits.

The gold standard co-functional gene links

Machine learning approaches for network construction requires gold standard linkage data. Pathway annotation databases are credible resources to collect them, yet current annotations for *C. neoformans* var. *grubii* (serotype A) are not sufficient. Therefore, we decided to use pathway annotations of *C. neoformans* var. *neoformans* (serotype D) because *C. neoformans* var. *neoformans* shares ~90% coding genome with *C. neoformans* var. *grubii* [16]. Pathway annotations by Kyoto Encyclopedia of Genes and Genomes (KEGG) were downloaded on August, 2013 [17], A total of 206,624 positive gold standard gene pairs were generated from 105 annotated KEGG pathways. To avoid network training bias toward a few dominant pathways, we excluded gene pairs derived from three over-dominant pathways [18]: metabolic pathways (cne:01100), biosynthesis of secondary metabolites (cne:01110), aminoacyl-tRNA biosynthesis (cne:00970). These three KEGG terms account for ~78 % of gold standard positive gene pairs. For orthology mapping between *C. neoformans* var. *grubii* and *C. neoformans* var. *neoformans*, we used an inparalog score threshold of 1 by Inparanoid (v4.1) [19]. The final gold standard set consists of 43,524 positive and 955,467 negative gene pairs among 1,414 *C. neoformans* genes.

Benchmarking and integrating networks

The likelihood of co-functional link supported by given experimental or computational data was measured by Bayesian statistics approach [20]. Log likelihood score (LLS) was calculated by following equation:

$$LLS = \ln\left(\frac{P(L|E)/P(\neg L|E)}{P(L)/P(\neg L)}\right)$$

, where $P(L|E)$ and $P(\neg L|E)$ are probability of gold standard positive and negative link for given experimental data, respectively, and $P(L)$ and $P(\neg L)$ are probability of gold standard positive and negative link before the experimental data provided, respectively. To avoid overtraining, we calculate LLS with 0.632 bootstrapping as described in [21].

For the links with multiple LLSs, we integrated the scores using weighted sum (WS) method as described in [21]:

$$\mathrm{WS} = L_0 + \sum_{i=1}^{n} \frac{L_i}{D \cdot i}, \; for\ all\ L \geq T,$$

, where L represents LLS (L_0 is the maximum LLS of a given functional link), and i is the index number for all other LLS by ranked order. D is a free parameter used as a weight factor, and T is a minimum threshold of LLS.

Co-functional links inferred from co-citation of genes (CC)

The original co-citation algorithm was based on an idea that functionally related two genes tend to be cited at the same research article abstract [22]. However, some articles have names of genes in the main text. To improve sensitivity of search, we scanned full text rather than just abstract for co-citation analysis. We searched PubMed Central (PMC, http://www.ncbi.nlm.nih.gov/pmc/) for articles containing "Cryptococcus neoformans" in abstract and any *C. neoformans* gene name in full text. As a result, we found a total of 609 articles containing *C. neoformans* gene names, and then assign probability of association between genes by Fisher's exact test.

Co-functional links inferred from co-expression of genes (CX)

We downloaded 12 microarray data sets containing no less than 8 samples of gene expression from Gene Express Omnibus (GEO) [23] on August, 2013. Pearson correlation coefficient was measured between all pairs of gene vectors of expression values to infer functional association between two genes. We tested a total of 12 microarray data sets and were able to infer functional links from seven of them: GSE31911 and 6 published data sets [24-29] including 191 samples (**Table S8**).

Co-functional links inferred from domain co-occurrence between proteins (DC)

Domains recur functional regions of proteins. Because domains are functional subunits of proteins, proteins that share a similar set of domains may contribute to a similar function. Using profiles of domain occurrence for proteins by InterPro database [30], we measure likelihood of functional association for given tendency of domain co-occurrence (DC) between two proteins. To learn a more informative co-occurrence pattern, we used a weighted version of the mutual information score, in which higher weights were given to rarer domains under the assumption that rarer domains harbor more specific pathway information.

Co-functional links inferred from genomic contexts (PG, GN)

We used genomic context information of *C. neoformans* proteins to discover functional associations between genes with two different methods, phylogenetic profiles [31-33] and gene neighbors [34-36]. The similarity of phylogenetic profiles between two *C. neoformans* genes reflects the degree of co-inheritance of two genes during speciation, because functional constraints between functionally coupled genes mainly determine co-inheritance pattern. We first ran BLASTP to compare all *C. neoformans* protein sequences against all protein sequences from 1,626 Bacteria genomes, 122 Archaea genomes, and 396 Eukaryota genomes. Phylogenetic profile matrices of the blast hit scores were constructed, and the similarity between profiles was measured by mutual information scores as described in [37]. For *C. neoformans* genes, we found that the similarity of phylogenetic profiles for each of the two domains of life (Archaea and Bacteria) performed better than that for all 2,144 genomes in retrieving gold-standard functional links. Therefore, we integrated the two domain-specific

networks into a single network by phylogenetic profiles. For network inference by genomic neighborhood across 1,746 prokaryote genomes, we used two approaches to measure the genomic neighborhood: chromosomal distance between neighboring genes [35,36,38] and probability of observed neighborhood [34]. Because we previously found complementarity between these two methods [39], we integrated them into a single network by genomic neighborhood.

Co-functional links by orthology-based transfer from yeast and human networks

Associalogs are conserved functional associations transferred from different species by orthology [21]. We transferred conserved functional links between *C. neoformans* genes from YeastNet v3 [40] and HumanNet [41]. All transferred co-functional associations are re-scored by Inparanoid weighted LLS (IWLLS) [42] as following,

IWLLS (A′-B′) = LLS (A-B) + ln(inparalog score of A-A′) + ln(inparalog score of B-B′)

,where A and B are *C. neoformans* genes and A′ and B′ are orthologous genes from *S. cerevisiae* or human, and the transferred functional association of A′-B′ from that of A-B obtains weighted values as how likely A-A′ and B-B′ are orthologous by Inparanoid. We transferred 7 and 2 co-functional associations of YeastNet v.3 and HumanNet, respectively (summarized in **Table S1**).

Assessment of prediction power of networks for pathogenicity

To assess predictive power of CryptoNet for *C. neoformans* pathogenicity, we used a total of 73 genes involved in three different virulence phenotypes were collected from literatures: 28 genes for capsule formation, 23 genes for melanin production, and 22 genes for thermotolerance. (**Table S2**). Predictions were performed for each of virulence phenotype. Ranks of virulence genes were assigned by sum of LLS by CryptoNet links from a gene to all other genes for the same virulence phenotype. If the known virulence genes are well interconnected by a given network, they are highly ranked by this network-assisted prioritizing method. The performance of network-assisted ranking was then assessed by the receiver operating characteristic (ROC) curve, which is usually summarized as a single score, area under the ROC curve (AUC). The ROC curve in general represents true positive (TP) rate for the given cost of false positive (FP) rate.

$$\frac{TP\ rate}{FP\ rate} = \frac{TP/TP + FN}{FP/FP + TN}$$

, where TP is a correctly predicted virulence gene, FP is an incorrectly predicted virulence gene, true negative (TN) is a correctly predicted non-virulence gene, and false negative (FN) is an incorrectly predicted non-virulence gene. AUC score ranges between 0.5 and 1, which indicate random prediction and perfect prediction, respectively. Visualization of networks for three virulence phenotypes were conducted by Cytoscape [43].

Prediction of novel candidate genes for anti-fungal drug resistance

To predict candidate genes for antifungal drug resistance, we devised a method of searching for context-associated hub genes. This method requires two components: subnetworks and an expression signature. Each subnetwork is composed of a hub gene having no less than 50

connected neighbors by CryptoNet and its neighbors. We predefined 2,135 subnetworks for the given number of neighbors as a threshold. An expression signature represents a cellular context such as drug stress condition. To construct an expression signature for antifungal drug stress, we collected 230 *C. neoformans* genes that were up-regulated by >2-fold upon treatment of fluconazole [44] (**Table S4**). Normalization of the expression data was conducted by Limma [45]. For the given pair of gene sets, one for 230 up-regulated genes by drug treatment and the other for neighbor genes of each of 2,135 hubs, we measured significance of enrichment using Fisher's exact test. If neighbors for a hub gene are significantly enriched among the up-regulated genes by fluconazole treatment, the corresponding hub gene is considered to be associated with the context of fluconazole treatment. We found 94 hub genes are significantly associated with gene expression response to fluconazole treatment (*p-value* < 0.05).

Construction of *ypk1*Δ, *sho1*Δ and *kin1*Δ mutants

The genes were deleted with a disruption cassette containing split Nat^r dominant selectable marker generated by double joint PCR as described before [46]. The gene disruption cassette was introduced into the H99S strain through the biolistic transformation method, as previously described [47]. The correct genotype of these mutants was confirmed by Southern blot analysis as described before [48]. Primers for this disruption and Southern blot analysis were described in **Table S9**.

Assay for virulence factor production, thermotolerance, and antifungal drug resistance

The *C. neoformans* Madhani collection strains used in this study were provided by Fungal Genetics Stock Center. Before tests, we confirmed the correct genotype of each strain by diagnostic PCR to check potential cross-contamination during strain storage and recovery. Each strain was cultured in a liquid YPD (yeast extract-peptone-dextrose) medium for 16 hours at 30°C. The agar-based DME (Dulbecco's modified Eagle's) medium (Invitrogen, Carlsbad, CA) for capsule production, and agar-based Niger seed medium, which contains indicated concentration of glucose (0.1% and 0.5%), for melanin biosynthesis were prepared as previously described [49]. The capsule production of strains showing growth defect at 37°C were determined at 30°C. The relative capsule size of each cell was quantitatively measured, previously described [49]. Melanin production was monitored and photographed daily. For thermotolerance test, strains were incubated overnight at 30°C in a liquid YPD medium, washed, serially diluted (1 to 10^4 dilutions) with dH_2O, and spotted onto a solid YPD medium. Each plate was incubated at 37°C or 39°C for 2-4 days and photographed for incubation period. For antifungal drug resistant test, each serially diluted strain (1 to 10^4 dilutions) was spotted onto a solid YPD medium containing the indicated concentration of azoles (fluconazole, itraconazole, and ketoconazole) or amphotericin B and incubated at 30°C and photographed for 2 to 5 days.

Galleria mellonella infection assay and *in vivo* mouse study

G. mellonella in the final instar larval stage (15 larvae per strain) was infected through prolegs with 800,000 *Cryptococcus* cells in 4 µl of PBS by the Hamilton syringe. After injection, larvae were incubated at 30 °C or 37°C for 14 days and then larvae showing signs, such as changes in body color and no movement in response to touch, were considered dead.

Larvae transforming into pupa were censored. The Kaplan-Meier survival curves were constructed by Prism 5.01 (GraphPad Software) and *p-values* were calculated from Gehan-Breslow-Wilcoxon test.

Four- to six-week-old female A/Jcr mice (National Cancer Institute, 18–20 g) were utilized in this study. For infection, strains were cultured in YPD medium overnight at 30°C, washed twice with phosphate buffered saline (PBS), and resuspended in PBS at 2×10^6 cells per ml. Serially diluted cells were plated onto YPD medium and incubated at 24°C for 72 hr to determine viability and CFU. Ten mice per strain (except 9 mice for strain YSB1720 due to one death after pentobarbital treatment) were anesthetized with pentobarbital (Lundbeck Inc.) and infected via intranasal instillation with 10^5 cells (in 50 µl). Survival was monitored twice daily, and moribund mice were CO_2-euthanized. The Kaplan-Meier survival curves were generated with Prism 5.02 (GraphPad Software), and P values were evaluated from a Log-rank (Mantel-Cox) test.

Ethics statement

The animal studies at Duke University Medical Center were in full compliance with the guidelines of the Duke University Medical Center Institutional Animal Care and Use Committee (IACUC) and the United States Animal Welfare Act (Public Law 98–198). The Duke University Medical Center IACUC approved all of the animal studies. The studies were conducted under protocol number A217-11-08 in Division of Laboratory Animal Resources (DLAR) facilities that are accredited by the Association for Assessment and Accreditation of Laboratory Animal Care (AAALAC).

Supplementary References

1 Rundlett, S. E. *et al.* HDA1 and RPD3 are members of distinct yeast histone deacetylase complexes that regulate silencing and transcription. *Proc. Natl. Acad. Sci. U. S. A.* **93**, 14503-14508 (1996).

2 Pijnappel, W. W. *et al.* The *S. cerevisiae* SET3 complex includes two histone deacetylases, Hos2 and Hst1, and is a meiotic-specific repressor of the sporulation gene program. *Genes Dev.* **15**, 2991-3004 (2001).

3 Liu, O. W. *et al.* Systematic genetic analysis of virulence in the human fungal pathogen *Cryptococcus neoformans*. *Cell* **135**, 174-188 (2008).

4 Haynes, B. C. *et al.* Toward an integrated model of capsule regulation in *Cryptococcus neoformans*. *PLoS Pathog.* **7**, e1002411 (2011).

5 O'Meara, T. R., Hay, C., Price, M. S., Giles, S. & Alspaugh, J. A. *Cryptococcus neoformans* histone acetyltransferase Gcn5 regulates fungal adaptation to the host. *Eukaryot. Cell* **9**, 1193-1202 (2010).

6 Roelants, F. M., Breslow, D. K., Muir, A., Weissman, J. S. & Thorner, J. Protein kinase Ypk1 phosphorylates regulatory proteins Orm1 and Orm2 to control sphingolipid homeostasis in *Saccharomyces cerevisiae*. *Proc. Natl. Acad. Sci. U. S. A.* **108**, 19222-19227 (2011).

7 deHart, A. K., Schnell, J. D., Allen, D. A. & Hicke, L. The conserved Pkh-Ypk kinase cascade is required for endocytosis in yeast. *J. Cell Biol.* **156**, 241-248 (2002).

8 Lee, H., Khanal Lamichhane, A., Garraffo, H. M., Kwon-Chung, K. J. & Chang, Y. C. Involvement of PDK1, PKC and TOR signalling pathways in basal fluconazole tolerance in *Cryptococcus neoformans*. *Mol. Microbiol.* **84**, 130-146 (2012).

9 Chabrier-Rosello, Y. *et al.* *Cryptococcus neoformans* phosphoinositide-dependent kinase 1 (PDK1) ortholog is required for stress tolerance and survival in murine phagocytes. *Eukaryot. Cell* **12**, 12-22 (2013).

10 Balciunas, D. & Ronne, H. Three subunits of the RNA polymerase II mediator complex are involved in glucose repression. *Nucleic Acids Res.* **23**, 4421-4425 (1995).

11 Chang, Y. W., Howard, S. C., Budovskaya, Y. V., Rine, J. & Herman, P. K. The rye mutants identify a role for Ssn/Srb proteins of the RNA polymerase II holoenzyme during stationary phase entry in *Saccharomyces cerevisiae*. *Genetics* **157**, 17-26 (2001).

12 Chen, C., Pande, K., French, S. D., Tuch, B. B. & Noble, S. M. An iron homeostasis regulatory circuit with reciprocal roles in *Candida albicans* commensalism and pathogenesis. *Cell Host Microbe.* **10**, 118-135 (2011).

13 Chen, C. & Noble, S. M. Post-transcriptional regulation of the Sef1 transcription factor controls the virulence of *Candida albicans* in its mammalian host. *PLoS Pathog.* **8**, e1002956 (2012).

14 Janbon, G. *et al.* Analysis of the genome and transcriptome of *Cryptococcus neoformans* var. *grubii* reveals complex RNA expression and microevolution leading to virulence attenuation. *PLoS Genet.* **10**, e1004261 (2014).

15 Cherry, J. M. *et al.* *Saccharomyces* Genome Database: the genomics resource of budding yeast. *Nucleic Acids Res.* **40**, D700-705 (2012).

16 Kavanaugh, L. A., Fraser, J. A. & Dietrich, F. S. Recent evolution of the human pathogen *Cryptococcus neoformans* by intervarietal transfer of a 14-gene fragment. *Mol. Biol. Evol.* **23**, 1879-1890 (2006).

17 Kanehisa, M., Goto, S., Sato, Y., Furumichi, M. & Tanabe, M. KEGG for integration and interpretation of large-scale molecular data sets. *Nucleic Acids Res.* **40**, D109-114 (2012).

18 Lee, I., Li, Z. & Marcotte, E. M. An improved, bias-reduced probabilistic functional gene network of baker's yeast, *Saccharomyces cerevisiae*. *PLoS One* **2**, e988 (2007).

19 Ostlund, G. *et al.* InParanoid 7: new algorithms and tools for eukaryotic orthology analysis. *Nucleic Acids Res.* **38**, D196-203 (2010).

20 Lee, I., Date, S. V., Adai, A. T. & Marcotte, E. M. A probabilistic functional network of yeast genes. *Science* **306**, 1555-1558 (2004).

21 Lee, I. *et al.* A single gene network accurately predicts phenotypic effects of gene perturbation in *Caenorhabditis elegans*. *Nat. Genet.* **40**, 181-188 (2008).

22 Stapley, B. J. & Benoit, G. Biobibliometrics: information retrieval and visualization from co-occurrences of gene names in Medline abstracts. *Pac. Symp. Biocomput.*, 529-540 (2000).

23 Barrett, T. *et al.* NCBI GEO: archive for functional genomics data sets--update. *Nucleic Acids Res.* **41**, D991-995 (2013).

24 Kim, M. S. *et al.* Comparative transcriptome analysis of the CO_2 sensing pathway via differential expression of carbonic anhydrase in *Cryptococcus neoformans*. *Genetics* **185**, 1207-1219 (2010).

25 Chun, C. D., Liu, O. W. & Madhani, H. D. A link between virulence and homeostatic responses to hypoxia during infection by the human fungal pathogen *Cryptococcus neoformans*. *PLoS Pathog.* **3**, e22 (2007).

26 Lee, H. *et al.* Cobalt chloride, a hypoxia-mimicking agent, targets sterol synthesis in the pathogenic fungus *Cryptococcus neoformans*. *Mol. Microbiol.* **65**, 1018-1033 (2007).

27 Ko, Y. J. *et al.* Remodeling of global transcription patterns of *Cryptococcus neoformans* genes mediated by the stress-activated HOG signaling pathways. *Eukaryot. Cell* **8**, 1197-1217 (2009).

28 Janbon, G. *et al.* Characterizing the role of RNA silencing components in

Cryptococcus neoformans. *Fungal Genet. Biol.* **47**, 1070-1080 (2010).

29 Bang, S., Lee, D., Kim, H., Park, J. & Bahn, Y. S. Global transcriptome analysis of eukaryotic genes affected by gromwell extract. *J Sci Food Agric* **94**, 445-452 (2014).

30 Hunter, S. *et al.* InterPro in 2011: new developments in the family and domain prediction database. *Nucleic Acids Res.* **40**, D306-312 (2012).

31 Huynen, M., Snel, B., Lathe, W., 3rd & Bork, P. Predicting protein function by genomic context: quantitative evaluation and qualitative inferences. *Genome Res.* **10**, 1204-1210 (2000).

32 Pellegrini, M., Marcotte, E. M., Thompson, M. J., Eisenberg, D. & Yeates, T. O. Assigning protein functions by comparative genome analysis: protein phylogenetic profiles. *Proc. Natl. Acad. Sci. U. S. A.* **96**, 4285-4288 (1999).

33 Wolf, Y. I., Rogozin, I. B., Kondrashov, A. S. & Koonin, E. V. Genome alignment, evolution of prokaryotic genome organization, and prediction of gene function using genomic context. *Genome Res.* **11**, 356-372 (2001).

34 Bowers, P. M. *et al.* Prolinks: a database of protein functional linkages derived from coevolution. *Genome Biol.* **5**, R35 (2004).

35 Dandekar, T., Snel, B., Huynen, M. & Bork, P. Conservation of gene order: a fingerprint of proteins that physically interact. *Trends Biochem. Sci.* **23**, 324-328 (1998).

36 Overbeek, R., Fonstein, M., D'Souza, M., Pusch, G. D. & Maltsev, N. The use of gene clusters to infer functional coupling. *Proc. Natl. Acad. Sci. U. S. A.* **96**, 2896-2901 (1999).

37 Date, S. V. & Marcotte, E. M. Discovery of uncharacterized cellular systems by genome-wide analysis of functional linkages. *Nat. Biotechnol.* **21**, 1055-1062 (2003).

38 Korbel, J. O., Jensen, L. J., von Mering, C. & Bork, P. Analysis of genomic context: prediction of functional associations from conserved bidirectionally transcribed gene pairs. *Nat. Biotechnol.* **22**, 911-917 (2004).

39 Shin, J., Lee, T., Kim, H. & Lee, I. Complementarity between distance- and probability-based methods of gene neighbourhood identification for pathway reconstruction. *Mol. Biosyst.* **10**, 24-29 (2014).

40 Kim, H. *et al.* YeastNet v3: a public database of data-specific and integrated functional gene networks for *Saccharomyces cerevisiae*. *Nucleic Acids Res.* **42**, D731-736 (2014).

41 Lee, I., Blom, U. M., Wang, P. I., Shim, J. E. & Marcotte, E. M. Prioritizing candidate disease genes by network-based boosting of genome-wide association data. *Genome Res.* **21**, 1109-1121 (2011).

42 Kim, E., Kim, H. & Lee, I. JiffyNet: a web-based instant protein network modeler for

newly sequenced species. *Nucleic Acids Res.* **41**, W192-197 (2013).

43 Smoot, M. E., Ono, K., Ruscheinski, J., Wang, P. L. & Ideker, T. Cytoscape 2.8: new features for data integration and network visualization. *Bioinformatics* **27**, 431-432 (2011).

44 Florio, A. R. *et al.* Genome-wide expression profiling of the response to short-term exposure to fluconazole in *Cryptococcus neoformans* serotype A. *BMC Microbiol.* **11**, 97 (2011).

45 Smyth, G. K. in *Bioinformatics and Computational Biology Solutions using R and Bioconductor* 397-420 (Springer, New York, 2005).

46 Kim, M. S., Kim, S. Y., Yoon, J. K., Lee, Y. W. & Bahn, Y. S. An efficient gene-disruption method in *Cryptococcus neoformans* by double-joint PCR with NAT-split markers. *Biochem. Biophys. Res. Commun.* **390**, 983-988 (2009).

47 Davidson, R. C. *et al.* A PCR-based strategy to generate integrative targeting alleles with large regions of homology. *Microbiology* **148**, 2607-2615 (2002).

48 Jung, K. W., Kim, S. Y., Okagaki, L. H., Nielsen, K. & Bahn, Y. S. Ste50 adaptor protein governs sexual differentiation of *Cryptococcus neoformans* via the pheromone-response MAPK signaling pathway. *Fungal Genet. Biol.* **48**, 154-165 (2011).

49 Bahn, Y. S., Hicks, J. K., Giles, S. S., Cox, G. M. & Heitman, J. Adenylyl cyclase-associated protein Aca1 regulates virulence and differentiation of *Cryptococcus neoformans* via the cyclic AMP-protein kinase A cascade. *Eukaryot. Cell* **3**, 1476-1491 (2004).
